# Translating research on seed dormancy and germination from Arabidopsis to temperate cereals to control pre-harvest sprouting

**DOI:** 10.3389/fpls.2026.1763984

**Published:** 2026-02-25

**Authors:** Renqiang Li, Muhammad Usama Hameed, Koen Geuten

**Affiliations:** Department of Biology, Leuven Plant Institute, Katholieke Universiteit Leuven, Leuven, Belgium

**Keywords:** Arabidopsis, Brachypodium, pre-harvest sprouting, seed dormancy, seed germination, temperate cereals

## Abstract

From slow, non-uniform germination to pre-harvest sprouting (PHS), both extremes of seed dormancy have posed challenges for plant breeders. Because this trait needs to be genetically tuned in relation to environmental cues, controlling the problem of pre-harvest sprouting can only be realized through a better understanding of the biological mechanisms of seed dormancy. Yet studying seed dormancy poses challenges, because of its complexity in the different modes of regulation (physical, chemical, developmental, physiological and genetic) in interaction with environmental cues (light, temperature, water and nutrients) and lack of natural variation in the commercial crop genetic resources. Building information from model systems can help guide our research efforts. While phylogenetically distant from temperate cereals, the available information for Arabidopsis is much more elaborate and can, to a certain extent, be translated. We therefore provide a comprehensive comparison of the mechanisms and pathways and indicate similarities, differences and knowledge gaps. While knowledge from Arabidopsis is highly valuable to guide seed dormancy studies in temperate cereals, effective knowledge translation that includes functional validation will often require the use of the more closely related “model system” Brachypodium. This model will also allow us to unravel derived or unique mechanisms in temperate cereals. As an indication of such derived mechanisms, we also discuss the genetic factors involved in seed dormancy control discovered in cereals, often through natural variation studies.

## 1 Introduction

When should a seed germinate? This question can decide the fate of a plant species. During evolution, species adapted to their surroundings and regulatory mechanisms emerged to ensure germination when the environment is favorable. These environmental monitoring mechanisms and decision responses collectively regulate germination timing through seed dormancy. Domestication interferes with this natural postponing mechanism as it aims for fast and uniform germination. The resultant accelerated germination in modern crop varieties results in reduced or complete lack of seed dormancy, inadvertently increasing susceptibility to pre-harvest sprouting (PHS), a phenomenon in which seeds germinate on the mother plant before harvest (Gubler et al., 2005). With climate change causing erratic rainfall during harvest seasons, PHS is becoming a substantial problem and leading to worldwide yield loss (Black et al., 2006). An effective solution could be to retrieve (part) of the original plant strategies, ideally a very strong, but quickly removable dormancy at maturity. To achieve this goal, a thorough understanding of seed dormancy and germination is indispensable.

Germination is defined as the sequence of physiological events that initiate with water uptake (imbibition) by the dry seed and conclude with radicle protrusion through the seed coverings (Bewley, 1997). If environmental conditions required for germination, such as ample moisture, optimum temperature, and oxygen, are not met, the seed does not germinate. This can be referred to as lack of germination rather than the presence of dormancy. These seeds can resume germination as the environment becomes favorable (Baskin and Baskin, 2004). In contrast, “seed dormancy refers to the inability of a viable seed to germinate even under otherwise favorable environmental conditions, due to an internal block” (Finch-Savage and Leubner-Metzger, 2006; Karssen et al., 1983). While the release of dormancy is affected by environmental signals, the genetic background of the plant controls the depth, rate of dormancy loss, and nature of environmental cues required to break dormancy. Thus, dormancy reflects a genetically programmed and environmentally modulated endogenous mechanism (Considine and Considine, 2016).

Dormancy enables seeds to monitor seasonal and temporal cues, assess competition, and sense nutrient availability, thereby ensuring the optimal timing to germinate. Furthermore, dormancy acts as a bet-hedging strategy to spread out germination timing, distance from mother plant, and maximize survival (Thompson and Ooi, 2010). Experimentally, seed dormancy levels can be evaluated through a germination test. Parameters like final germination percentage or time-integrated matrices, such as germination index, are commonly used (Strand, 1980). This is why experiments need to be carefully designed and interpreted to distinguish the two processes. In the laboratory, primary seed dormancy can be assessed using freshly harvested seeds, while seed germination can be tested using after-ripened (non-dormant) seeds (Bethke et al., 2004).

Though seed dormancy can be categorized in different ways, five major classes have been distinguished and widely utilized, namely morphological, physiological, physical, morphophysiological, and combinational dormancy (Baskin and Baskin, 2004). In physiological dormancy, mature seeds stay dormant by internal physiological and metabolic restrictions within the embryo, so-called embryo-imposed dormancy, rather than the seed coat or underdeveloped seed structures. In temperate cereals and Arabidopsis, only physiological dormancy is relevant, which constitutes the focus of this manuscript (Bewley, 1997). Physical dormancy is caused by one or more water-impermeable layers of palisade cells in the seed or fruit coat (Baskin and Baskin, 2004). Although seed coverings and seed coat in temperate cereals can influence germination by interfering with gas exchange, namely coat-imposed dormancy, they do not cause true physical dormancy (Simpson, 1990).

Based on the timing of dormancy induction, it can be primary or secondary. Primary dormancy is established within the mother plant during seed development (Karssen et al., 1983). At physiological maturity, seed dormancy levels may range from deeply dormant to fully nondormant (Bewley, 1997). After dispersal, primary dormancy can be lost gradually, but afterwards, seeds may acquire secondary dormancy if they experience long-term unfavorable environmental conditions, such as hypoxia or extreme temperatures. Notably, secondary dormancy can only be induced with some residual primary dormancy, emphasizing the hierarchical relationship (Baskin and Baskin, 2004).

Studying seed dormancy in cultivated cereals is not always straightforward, mainly due to the absence of natural variation because of long-term artificial selection for reduced dormancy (Tai et al., 2021). The complex genomes of cereal crops make it even harder to tap into these genetic resources. Furthermore, cereals are often recalcitrant to genetic transformation, hindering the effective application of available functional genomic tools (Chen et al., 2022). Using a closely related “crop model” with similar growth conditions can accelerate research on this trait. *Oryza sativa* (rice) could be a practical option for investigating seed dormancy in tropical cereals, but it is not ideal for temperate cereals due to different growth conditions (Fujino et al., 2004). A model system like *Brachypodium distachyon* carries unique opportunities with abundant resources and tools (Mur et al., 2011). Although research on seed dormancy in Brachypodium is still in its infancy, previous discoveries in Arabidopsis can be a good starting point for translation to temperate grasses (Scholthof et al., 2018).

*Arabidopsis thaliana* has been instrumental in advancing our understanding of plant developmental mechanisms, including seed dormancy. Extensive research on this model organism has elucidated the genetic and environmental regulation of dormancy by factors such as light, nitrogen, temperature, and phytohormones (Bethke et al., 2006; Footitt et al., 2013; Hilhorst and Karssen, 1988). These studies provided important insights into dormancy mechanisms, such as major ABA and GA metabolism genes, hormonal signaling pathways and key dormancy regulators like *DELAY OF GERMINATION 1* (*DOG1)* (Sajeev et al., 2024). However, due to evolutionary diversification in morphology, physiology and ecology, translating findings from Arabidopsis to the economically important temperate cereals faces multifaceted challenges (Roeder et al., 2025; Uauy et al., 2025).

In this review, we provide a comparative overview of dormancy and germination regulation between Arabidopsis and representative temperate cereals. These include the conserved central hormone balance between ABA and GA, the key player *DOG1*, and environmental regulation of seed dormancy induction and release, while also bringing up recent progress in epigenetic regulation of seed dormancy and PHS resistance. By highlighting shared and species-specific genetic pathways, we aim to elucidate areas that can be adapted from existing knowledge, while also pointing out gaps and opportunities that warrant further investigations. As such, we explore the potential use of these genetic pathways in addressing the problem of pre-harvest sprouting.

## 2 Pre-harvest sprouting in temperate cereals and the potential role of Brachypodium research

As an emerging problem, PHS stems from a lack of grain dormancy and is under strong environmental influence, most notably rainfall, temperature, and humidity (Tai et al., 2021). PHS induces precocious seed germination by shifting the hormonal balance, resulting in cellular and oxidative damage, reduced desiccation tolerance and increased susceptibility to pathogens, hence affecting seed viability (Black et al., 2006; Espinosa-Ramírez et al., 2021). During harvest season, sprouting on the mother plant initiates embryo-driven reserves mobilization through enzymes like α-amylase and proteases, leading to starch and protein breakdown in the endosperm (Matilla, 2024). In wheat, elevated α-amylase activity caused by PHS degrades starch into smaller sugars, lowering the Hagberg falling number, which is a measure of α-amylase activity through dough viscosity (a low falling number means high enzyme activity). This results in dense and gummy bread textures, rendering the grain unsuitable for baking. Partial sprouting, though not visible, can still lead to similar enzymatic effects. Furthermore, reduced viability disqualifies the grain from being used as a seed. Consequently, PHS-affected wheat is typically relegated to feed quality (Olaerts and Courtin, 2018). Barley, bred for uniform and early germination for malting, faces even more severe challenges because even mild PHS can drastically reduce grain quality. Partial cell wall breakdown during malting also increases β-glucan levels, leading to cloudy beer and industrial filter clogging. Like wheat, barley also suffers yield losses, and high moisture in sprouted seeds elevates the risk of fungal infection (Rooney et al., 2023).

**Figure 1:**
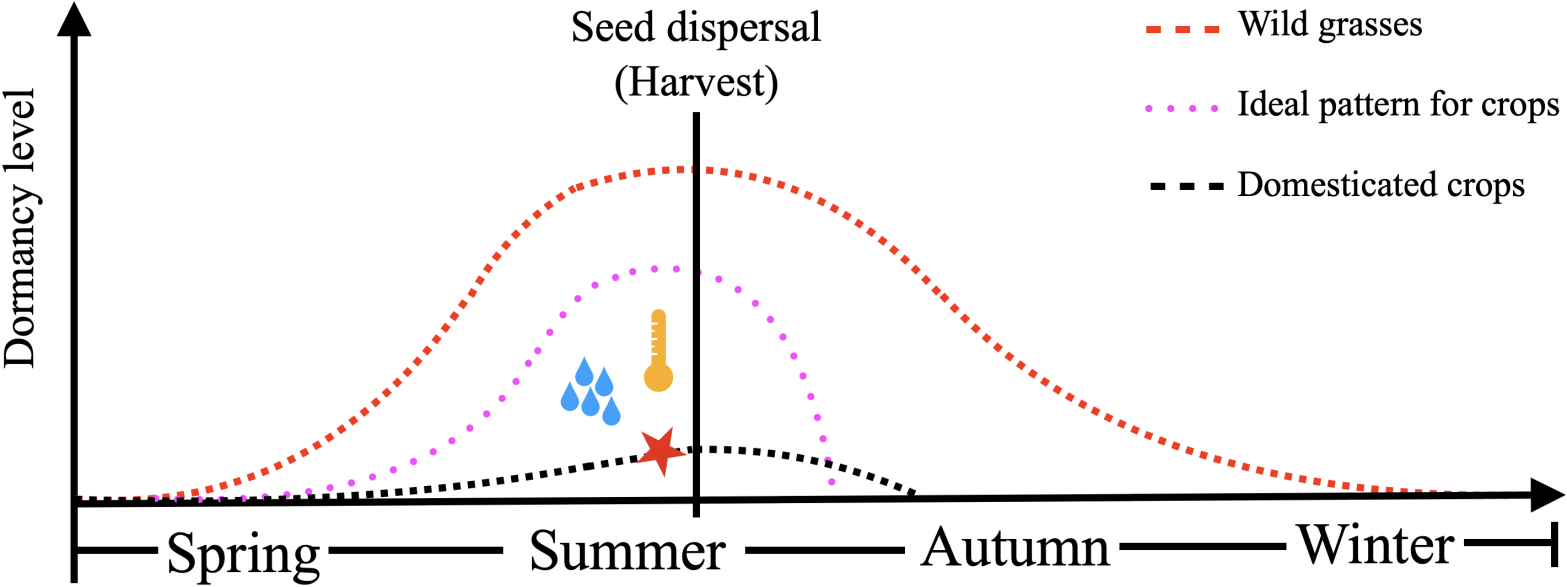
Seasonal seed dormancy fluctuation of undomesticated wild grasses (red line), ideal seed dormancy pattern for temperate cereals (pink line) and domesticated modern cereal crop cultivars (black line). Wild grasses show relatively deeper seed dormancy at the time of seed dispersal and lose dormancy gradually. Modern cereal crop cultivars show very shallow seed dormancy throughout their life cycle due to artificial domestication. The ideal seed dormancy pattern for temperate cereals features an intermediate level of seed dormancy, which peaks at harvest. This dormancy can be released shortly (~2 weeks for barley, ~4 weeks for wheat) post-harvest.

Maintaining a manageable level of dormancy in commercial cereals offers a potential solution to PHS. A controllable switch from dormancy to germination, such as time-based after-ripening, is highly desirable (Rodríguez et al., 2015). A different “switch” in the form of a low-cost chemical or environmental treatment to trigger quick dormancy release could also be useful. While agronomic practices, like induced drought and variable nitrogen dosing, have been reported to be effective in enhancing PHS resistance, breeding for moderate dormancy remains the most sustainable approach for resistance (Biddulph et al., 2005; Yang et al., 2025b). The optimal dormancy level varies based on cereal species and end-use. In wheat used for baking, deep dormancy does not hinder industrial use, though it interferes with use as seed. For bread wheat intended for seed, moderate dormancy at physiological maturity, requiring 4–6 weeks of after-ripening, is ideal. Conversely, for industrial malting barley, very low dormancy is essential. Maltsters require high germination rates and speed, as any residual dormancy raises operational costs. The ideal dormancy pattern for barley should resist PHS while being malting-compatible, with a short after-ripening period (1–2 weeks) and uniform germination capability (Woonton et al., 2005). Other cereals, such as oats and rye, generally exhibit stronger dormancy (Simpson, 1990). Overall, similar principles apply that low dormancy or rapid dormancy loss is needed for germination based industrial processing, while uniform field germination is crucial for seeding establishment in agriculture. Thus, a relatively high dormancy with a quick controllable loss of dormancy would be ideal for commercial cereals (**Figure 1**).

To achieve this ideal dormancy pattern, the temperate grass model plant *Brachypodium distachyon* (purple false brome) offers unique advantages. It has a small diploid genome (~300 Mb), a short life cycle, and close physiological resemblance to temperate grasses. Moreover, it is self-pollinated, short-statured, and easily transformable, making it ideal for functional studies (Alves et al., 2009; Brkljacic et al., 2011). For seed dormancy and germination study, Brachypodium, as an undomesticated wild grass, could be superior to commercial varieties, owing to its extensive variation in seed dormancy and germination timing (Barrero et al., 2012; Kosina and Tomaszewska, 2016). Anatomical studies indicate a shared spikelet style of floral structure between Brachypodium and temperate cereals (Barrero et al., 2012; Opanowicz et al., 2008). Furthermore, Brachypodium seeds respond to dormancy-imposing and dormancy-breaking environmental conditions like temperate cereals. The effect of maternal temperature, light and after-ripening on Brachypodium seed dormancy has been characterized, showing consistent behaviors across grass species (Barrero et al., 2014, 2012; Elgabra et al., 2019; Li et al., 2019; Vidaller et al., 2018). Finally, Brachypodium grains contain most cell wall polysaccharides found in other cereal grains (Guillon et al., 2011; Hands and Drea, 2012). The roles of husks and membranes, anatomical observations of coleorhiza and embryo behavior during the early stages of grain germination have been extensively explored by independent research (Barrero et al., 2012; El-Keblawy et al., 2019; Wolny et al., 2018). Notably, endo-beta-mannanase and cathepsin B-like protease, known influencers of Arabidopsis and tomato seed germination, have been shown to play similar roles during wheat, barley and Brachypodium grain germination (Gonzalez-Calle et al., 2015; Iglesias-Fernández et al., 2019; Isabel-LaMoneda et al., 2003). In addition, epigenetic modifications have also been examined during Brachypodium grain germination, which may provide resources to develop comparable insights when integrated with data from wheat and barley (Gao et al., 2012; Kapazoglou et al., 2010; Wolny et al., 2017). Given its physiological similarity to temperate cereals and genetic tractability, *Brachypodium distachyon* holds the potential of a powerful model for seed dormancy research, which could accelerate the discovery of functional genes and their regulatory mechanisms, ultimately aiding breeding efforts for PHS resistance in wheat, barley, and other temperate cereals. However, more intensive investigation would be anticipated to deepen our understanding of seed dormancy and germination regulation and assist knowledge translation into economically important temperate cereals.

## 3 Seed dormancy regulation through the seed coat and coverings

Seed morphology is an important factor in the regulation of seed dormancy. A typical desiccation-tolerant or orthodox seed consists of a combination of living and dead tissues. Dead tissues mainly contribute to physically preventing the seed from germinating through specialized structures and hardened seed coats or seed coverings. They can influence germination by interrupting water or oxygen uptake or by physically blocking the embryo from emerging. In addition, certain molecules present in the testa or husk can regulate dormancy.

**Figure 2:**
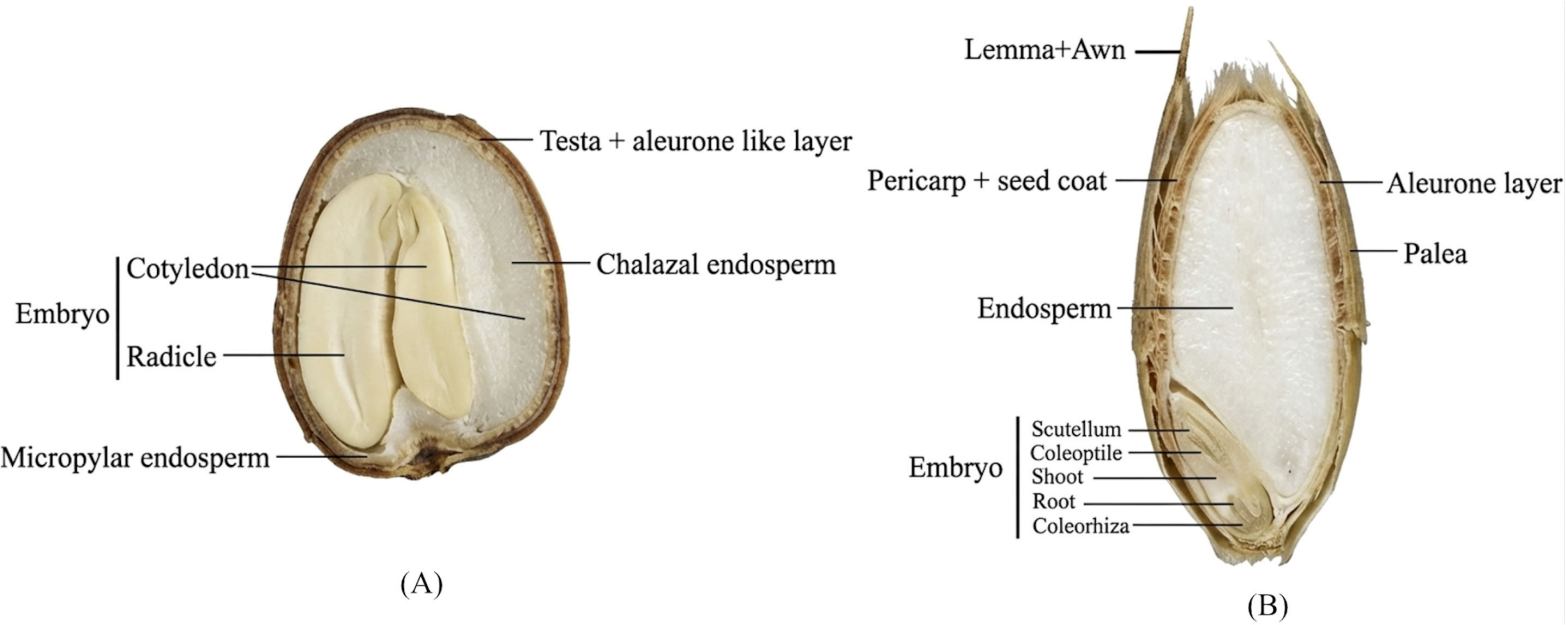
Schematic drawing of seeds from grasses and Arabidopsis. Seed structure and nomenclature based on published reviews (Finch-Savage and Leubner-Metzger, 2006; Rodríguez et al., 2015). **(A)** An Arabidopsis seed. Embryo dominates seed volume while the surrounding endosperm, including the chalazal and micropylar ones, contributes a minimal amount. No external coverings are present. The image was generated using Gemini (Google) based on author-provided prompts and subsequently reviewed and edited by the authors. Figure not drawn to scale. **(B)** A typical floret from temperate grasses. Embryo, showing coleorhiza, root, shoot, coleoptile and scutellum, accounts for a minimal percentage of the mature seed composition, while the starchy endosperm contains most seed reserves. Lemma and Palea are also present outside.

In Arabidopsis seeds, the ovule integuments form the dead testa (seed coat), but unlike cereals, they lack a husk (**Table 1**). During seed maturation, the outer layer accumulates a waxy cuticle, while the inner membrane develops different pigments such as flavonoids (proanthocyanidins) and tannins (**Figure 2A**). The presence of these pigments creates a tight hydrophobic layer that affects oxygen and water uptake during imbibition. Additionally, Arabidopsis seed coats accumulate the fatty polymer suberin, which also interferes with oxygen exchange (Fedi et al., 2017). Considering the involvement of protein and mRNA oxidation in after-ripening and germination, the link between these processes and the seed barrier has been extensively characterized (El-Maarouf-Bouteau et al., 2013). Experimentally, water uptake is tested using dyes such as tetrazolium. In non-dormant seeds with more permeable testa, higher water uptake was observed. Scarification (physical damage to the seed coat) can increase germination in deeply dormant seeds. Consistent with this, Arabidopsis *transparent-testa* (*tt*) mutants—*tt2* (encoding an R2R3 MYB domain protein), *tt4* (encoding chalcone synthase), and *ttg1* (encoding a WD-repeat-containing protein)—produce seeds lacking proanthocyanidins and germinate faster than wild-type controls (Debeaujon et al., 2000). Moreover, suberin and flavanols have been found to modify seed coat structure through temperature-dependent lignification (Hyvärinen et al., 2025; MacGregor et al., 2015). Low temperature during mother plant development promotes lignification and suberization of a polar lignin barrier in the outer integument cells of seeds. Transcription factors MYB9 and MYB107 were confirmed to be responsible for these modifications, with predominant contribution from MYB107 under cold temperature and a lesser role played by MYB9 (Hyvärinen et al., 2025).

**Table 1:** A comparative overview of seed structure and environmental regulation of seed dormancy in Arabidopsis and temperate cereals.

Aspect	Arabidopsis	Temperate cereals
Seed characteristics		
Seed Area	~0.1 – 1.1 mm^2^	~4–25 mm² depending on species
Type	Eudicot	Monocot
Seed living structures	Embryo, endosperm	Embryo, aleurone layer in endosperm
Dead Parts	Testa	Testa and pericarp; in hulled cereals (barley, oat) lemma and palea remain adherent
Embryo proportion	Embryo covers the major proportion of seed	Small embryo with large endosperm
Endosperm Storage	Minimal; nutrients are mainly stored in cotyledons, mainly fat and protein	Primary nutrient reservoir for germination; large starchy endosperm characteristic of temperate cereals
Reserve mobilization	Quick; upon germination	Slower; after reserve breakdown
Seed Type	Orthodox (High desiccation resistance)	Orthodox (High desiccation resistance)
Seed coat & coverings	Seed coat derived from two integuments, with cutin layers, imposes biochemical dormancy	In naked cereals (wheat, rye), testa–pericarp coverings impose biochemical/physical dormancy In hulled cereals (barley, oat), lemma and palea tightly adhere and add mechanical and oxygen-limiting constraints
Embryo	Embryo growth is repressed by signals from endosperm and seed coat	Embryo growth is constrained by coverings; embryo-derived GA activates aleurone for reserve mobilization and interacts with ABA signaling
Dormancy induction and environmental regulation		
Type of dormancy	Physiological	Physiological
Primary dormancy establishment	Dormancy establishment during seed development and pre-anthesis	Dormancy is established during seed development and pre-anthesis
Secondary dormancy	Non−dormant imbibed seeds can re−enter dormancy under unfavorable conditions	Temperate cereals can enter secondary dormancy under unfavorable conditions (e.g., high temperature, low oxygen); mechanisms involve increased ABA sensitivity and sometimes ABA synthesis
After-ripening	Weeks to months	Few weeks to a year, depending on domestication and species
Stratification (cold)	Effective: imbibition at low temp (e.g. 4 °C) breaks dormancy	Cold, moist stratification (e.g., 4 °C) effectively breaks dormancy in temperate cereals
Temperature effects	Low maturation temperature → deeper dormancy. Cold imbibition → germination. High imbibition temp → Dormancy	Cool maturation temperatures increase dormancy; warm maturation reduces it. High imbibition temperatures cause thermoinhibition and longer exposure can promote secondary dormancy
Light effects	Germination often requires light Red light strongly stimulates germination	Optimal germination in darkness Red light enhances germination in Brachypodium Blue and white light commonly inhibit germination in barley and wheat
Hormonal control		
Hormonal Balance	ABA/GA balance is central High ABA in endosperm maintains dormancy; GA promotes germination Endosperm regulates embryo hormone levels	ABA/GA balance central Dormancy variation largely reflects ABA sensitivity in the embryo and coverings Aleurone responds to GA to initiate reserve mobilization
ABA Metabolism	Extensively characterized, including biosynthesis and catabolism	Key ABA metabolic genes identified (e.g., NCED, ABA8’OH), but regulatory networks remain less resolved than in Arabidopsis
Sensitivity to Hormones	Dormancy and germination highly sensitive to ABA/GA ratio	Hormone sensitivity, especially to ABA, is a major determinant of dormancy depth Increased GA sensitivity leads to germination
Additional Hormones	Ethylene, brassinosteroids, cytokinin, and salicylic acid play supporting roles	Roles for ethylene, brassinosteroids, cytokinins, and salicylic acid are supported but species- and stage-dependent
Dormancy Depth	High variation of seed dormancy, depending on ecotypes and accessions	A range of dormancy depending on domestication or not; wild accessions and landraces show deep dormancy

Besides seed coats, Arabidopsis endosperm contributes to seed dormancy significantly, though it is very small in size compared to embryos and does not contain major storage compounds (Bethke et al., 2007). However, the single-celled aleurone-like-layered endosperm plays crucial roles in regulating seed dormancy through phytohormone signaling. The Arabidopsis endosperm maintains dormancy by providing ABA-rich hormonal suppression, restricting GA signaling, resisting radicle emergence, and modulating embryo growth through maternal and gene-regulatory signals (Doll and Ingram, 2022). During seed development, the endosperm-expressed transcription factors *ZHOUPI* and *INDUCER OF CBF EXPRESSION 1* determine the depth of primary seed dormancy in Arabidopsis (MacGregor et al., 2019). Upon seed imbibition, endosperm responds to nitric oxide, GA and ABA. *ABI5* expression in the endosperm defines altered and spatially distinct ABA signaling in contrast to *ABI4* expression confined to the embryo (Penfield et al., 2006b). In addition, endosperm controls seed germination via mannanase mediated radicle emergence and release or transport of ABA into the embryo (Iglesias-Fernández et al., 2011; Kang et al., 2015; Lee et al., 2010; Leubner-Metzger, 2002).

The cereal grain (caryopsis) has a thin testa fused to the pericarp. The lemma and palea (collectively called the husk) enclose the seed, either loosely (e.g., wheat) or tightly (e.g., barley, Brachypodium). In cereals, the seeds are relatively large with a single cotyledon (**Figure 2B**). The combined outer coverings (testa and husk) play a major role in coat-imposed dormancy, similar to Arabidopsis. These structures accumulate various phenolic compounds, including phenolic acids, coumarins, tannins, and flavonoids. Many of these compounds have been shown to strongly inhibit germination (Rusu et al., 2023). One important chemical group is the flavan-4-ols, precursors of phlobaphenes responsible for red coat color in wheat, barley, and rice (Groos et al., 2002; Himi et al., 2005). Notably, the wheat *R-1* locus (also called *PHS-3D*) encodes a MYB-type transcription factor named *Tamyb10*, which controls husk and coat pigmentation (Himi and Noda, 2005; Lang et al., 2021). Overexpression of *Tamyb10-D* in the white-grained wheat cultivar Fielder led to red-grained seeds showing significantly delayed germination, which correlated with higher flavonoid and ABA production contributed by upregulated expression level of genes in the flavonoid biosynthesis pathway and ABA biosynthesis pathway (Lang et al., 2021). In rice, the *RED COLEOPTILE LOCUS* (*OsRc)* controls seed dormancy and pigmentation by regulating ABA and flavonoid biosynthetic pathways, respectively (Gu et al., 2011). Phylogenetic analysis revealed high sequence similarity between *OsRc*, *Tamyb10*, the barley proanthocyanidin synthesis locus (*Ant28*; candidate gene *HvMYB10*), and the *Arabidopsis TRANSPARENT TESTA (TT)* genes (Furukawa et al., 2007).

In temperate cereals, husks can hinder oxygen uptake and enhance dormancy; their removal may therefore reduce dormancy (Barrero et al., 2012; Bradford et al., 2008). Although the husk remains tightly attached in barley, it plays a particularly significant role. It has also been suggested that the husk functions as a light filter, allowing only certain wavelengths to pass and thus affecting germination. At least in wheat, water movement did not differ significantly between dormant and non-dormant seeds, ruling out reduced water availability as the main reason for husk-imposed dormancy (Rathjen et al., 2009). Instead, oxygen uptake is the key factor, as shown by the fact that dormant barley seeds can germinate more readily in a high-oxygen environment (Bradford et al., 2008). Phenolic compounds in the husk likely serve as substrates for oxidation reactions, creating a low-oxygen atmosphere and reducing respiration in the aleurone layer and embryo. Oxygen uptake in temperate cereals is also temperature-regulated: under 15 °C incubation, oxygen content beneath the husk may rise to 15.8%, whereas at 30 °C it can drop to 0.3% (Hoang et al., 2014). After-ripened grains show a reduced effect of husk-imposed dormancy, even though they do not exhibit significant differences in phenolic compound composition (Rodríguez et al., 2015).

Endosperm in cereals is a large persistent tissue and contains major storage molecules with a surrounding aleurone layer. The large endosperm occupies most of the grain volume (Barrero et al., 2012; Kesavan et al., 2013). At maturity, the endosperm is mostly starchy and functions as a dead storage tissue, with a living single-celled aleurone layer (Liang et al., 2025). Unlike Arabidopsis, which consumes most of its endosperm during embryogenesis, cereals retain the endosperm as the primary storage tissue. The aleurone layer acts as a major regulator of dormancy by maintaining high ABA sensitivity and upregulating the ABA responsiveness genes in dormant seeds, while also functions as a signaling component for the embryo (Matilla, 2024). Moreover, cell wall modification and α-amylase activation occur through the aleurone layer (Hedden, 2025).

Additionally, studies in grasses, including barley, Brachypodium and oat, have shown that the coleorhiza tissue plays a pivotal role in causing dormancy and preventing germination, mainly through inhibiting ABA catabolism and affecting cell wall modification (Barrero et al., 2009; Gonzalez-Calle et al., 2015; Holloway et al., 2021; Millar et al., 2006). Coleorhiza hairs developed on rice embryo surfaces have also been implicated in grain germination, but through a distinct mechanism related to atmospheric moisture uptake (Bin Rahman et al., 2022).

Overall, seed covering structures can play a major role in both temperate cereals and Arabidopsis for dormancy regulation, although the influence is generally more prominent in cereals due to the presence of husks. With respect to PHS, dormancy regulation through the husk is particularly relevant. Coat/husk-imposed dormancy interacts with embryo-regulated dormancy to determine the final dormancy level. Later sections will discuss embryo-regulated dormancy in more detail.

## 4 ABA and GA as a central conserved mechanism of dormancy and germination

### 4.1 ABA and GA hormone balance in seed dormancy and germination

The hormone balance theory suggests that ABA and GA act antagonistically to regulate seed dormancy and germination (Yamaguchi et al., 2007). Genetic analysis of hormone metabolism and signaling mutants clearly showed that a mutant deficient in metabolism or signaling of one hormone can be complemented by a mutation in the other (Debeaujon and Koornneef, 2000; Koornneef et al., 1982; Steber et al., 1998; White et al., 2000; Xian et al., 2024). Under dormancy-promoting conditions, an increased ABA/GA ratio was consistently observed, while reduced ratios were observed under dormancy breaking conditions (Finch-Savage et al., 2007; Kendall et al., 2011; Liu et al., 2010; Toh et al., 2008).

**Figure 3:**
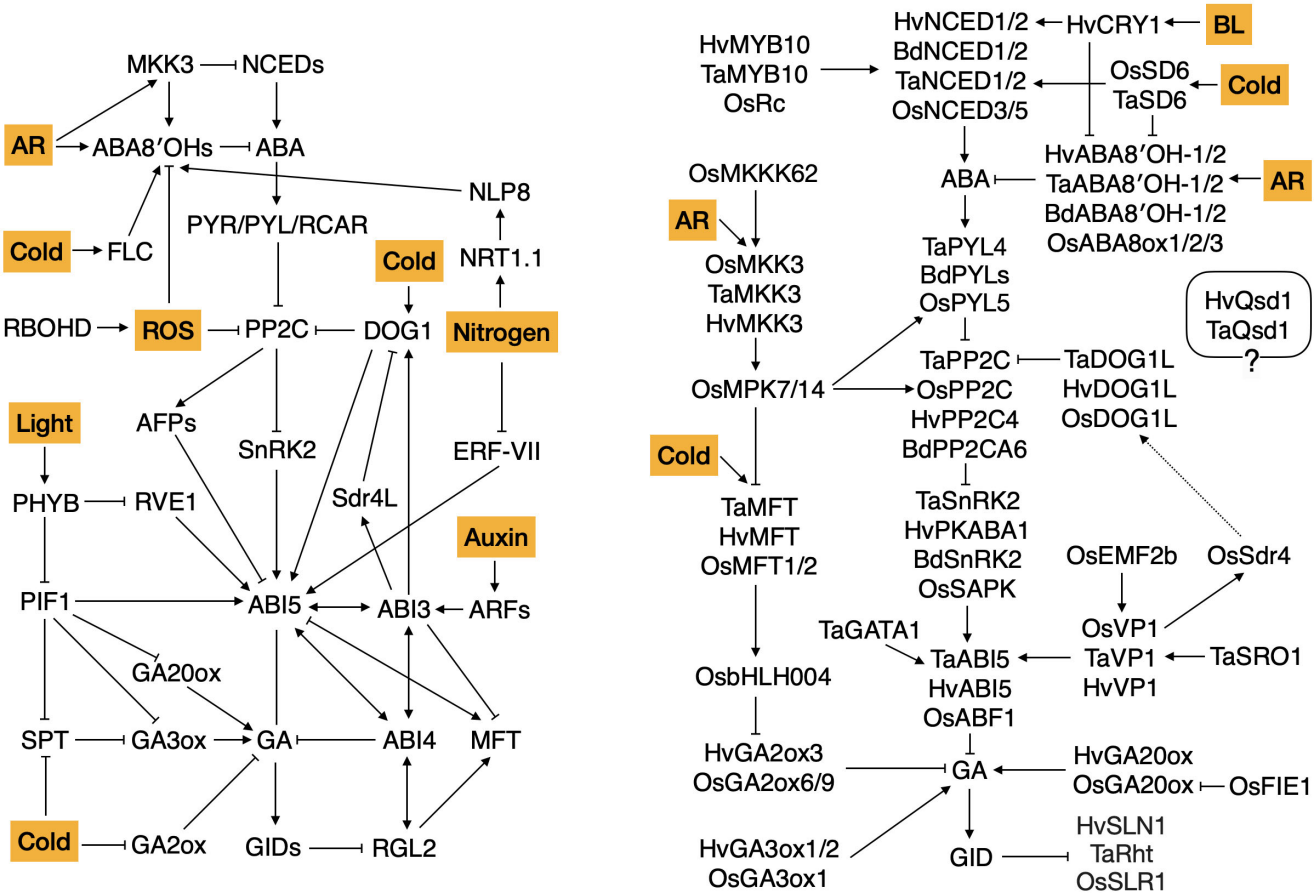
Genetic pathways controlling seed dormancy in Arabidopsis and cereals. The arrow indicates promoting effect; T-bar indicates inhibiting effect. Environmental and internal cues are indicated by color boxes. ABA, abscisic acid; GA, gibberellic acid; AR, after-ripening; ROS, reactive oxygen species; BL, blue light. Identifiers show the species as follows: At for Arabidopsis thaliana, Bd for Brachypodium distachyon, Os for Orzya sativa (rice), Hv for Hordeum vulgare (barley), Ta for Triticum aestivum (wheat). The complete nomenclature of proteins can be found in the text. The pathways were compiled from the following reviews (Bewley, 1997; Dong et al., 2022; Finch-Savage and Leubner-Metzger, 2006; Finkelstein et al., 2008; Graeber et al., 2012; Holdsworth et al., 2008; Iwasaki et al., 2022; Koornneef et al., 2002; Penfield and MacGregor, 2016; Rodríguez et al., 2015; Sano and Marion-Poll, 2021; Shu et al., 2016; Tai et al., 2021; Tuan et al., 2018) and research articles cited in the text. The central ABA and GA hormonal pathways are fundamental in seed dormancy induction, maintenance and release in Arabidopsis and cereals. ABA metabolism occurs through biosynthesis and catabolism components, with NCEDs and CYP707As playing key roles, respectively. ABA perception by PYR/PYL/RCAR receptors deactivates PP2Cs, which relieves its inhibition on SnRK2, leading to the activation of downstream targets, including key transcription factorsABI3, ABI4 and ABI5. Bioactive GAs are synthesized by GA-20 oxidases and GA-3 oxidases primarily and can be deactivated by GA 2-oxidases. GA binding to the GID receptor releases the inhibition on germination imposed by DELLA proteins (showing the major germination repressor RGL2 for Arabidopsis and other DELLA homologues in cereals), thus favoring cell wall loosening and seed germination. Left panel, in Arabidopsis, the central hormonal pathways integrate diverse environmental cues, including temperature, light and nutrients. This integration involves hierarchical signal transductions from environmental sensors, intermediate transducers, to hormonal signaling components. Right panel, in cereals, major seed dormancy regulators, including MKK3, MFT, Sdr4 and DOG1, control this trait through hormonal pathways. The major barley seed dormancy locus Qsd1 has not been linked to the hormonal pathway and is thus marked by a question mark. Regulation of ABA and GA is well studied in both systems, but genetic regulation through different environmental signals is still missing in cereals.

Research across various species supports the fundamental roles of ABA and GA, showing that ABA promotes seed reserve accumulation, dormancy induction, and desiccation tolerance, while GA facilitates germination, subsequent seedling establishment and growth (Garciarrubio et al., 1997; Penfield et al., 2006a; Shu et al., 2018). Abscisic acid levels are determined by the net outcome of biosynthesis and catabolism. In Arabidopsis, *9-CIS-EPOXYCAROTENOID DIOXYGENASE (NCED)* enzymes, encoded by a family of five genes, have been proven to be key in ABA synthesis, especially *AtNCED6* and *AtNCED9* (Lefebvre et al., 2006; Tan et al., 2003). *AtCYP707A1* and *AtCYP707A2*, which encode ABA 8′-hydroxylases, participate in the ABA catabolism pathway but function at different developmental stages and are indispensable for proper seed dormancy and germination control, while *AtCYP707A3* displays partial functional redundancy based on the analyses of *cyp707a* triple mutant (Kushiro et al., 2004; Okamoto et al., 2006). Besides hormonal content, ABA signaling is also critical for seed dormancy. Core ABA signaling components include ABA receptors, clade A protein phosphatase 2Cs (PP2Cs), and SNF1-related protein kinase 2 (SnRK2) proteins (Cutler et al., 2010). Upon ABA perception, suppressed PP2C activity leads to the activation of SnRK2, which modulates downstream targets including the B3 domain-containing transcription factor ABSCISIC ACID INSENSITIVE3 (ABI3), the AP2 domain-containing transcription factor ABI4 and the bZIP transcription factor ABI5 (**Figure 3**).

Opposite to ABA, GA is involved in germination under favorable environmental conditions. In Arabidopsis, gibberellin 20-oxidase (GA20ox) and gibberellin 3-oxidase (GA3ox) are the major components of GA biosynthesis, while gibberellin 2-oxidase (GA2ox) is responsible for catabolism (Yamaguchi et al., 2007; Yamauchi et al., 2004). Gibberellic acid affects seed dormancy through signaling components including GIBBERELLIN INSENSITIVE DWARF1 (GID1), DELLA proteins and SLEEPY1 (SLY1). GA binding to GID1 receptors induces the degradation of DELLA proteins, which occurs through a ubiquitin–proteasome pathway involving the F-box-containing protein SLEEPY1 (SLY1), leading to the de-repression of germination imposed by DELLA proteins, including gibberellic-acid insensitive (GAI), repressor of ga1-3 (RGA), RGA-LIKE 1 (RGL1), and RGA-LIKE 2 (RGL2) (**Figure 3**) (Dill et al., 2004; Sun, 2011). Loss of function of *GAI*, *RGA*, *RGL1* and *RGL2* enables GA deficient *ga1–3* mutant seeds to germinate in a wide range of environments (Cao et al., 2005), while mutations in *SLY1* lead to increased seed dormancy and ABA sensitivity during germination (Steber et al., 1998). These observations suggest that GA signaling components integrate both environmental and endogenous cues for the seed to germinate.

### 4.2 Conservation of ABA and GA regulated seed dormancy in temperate cereals

Sequence similarity searches have uncovered major hormonal metabolism and signaling genes in temperate cereals, including wheat, barley and Brachypodium (Barrero et al., 2012; Chono et al., 2006; Son et al., 2016; Zhang et al., 2014). These include *NCEDs, CYP707As* relating to hormone metabolism, and major signaling genes such as *ABI3* and *SnRK2* (Shu et al., 2016). Besides sequence similarities, a clear link exists between gene expression and hormonal level which also correlate with grain dormancy and germination phenotypes. In wheat, barley, Brachypodium and rice, mechanisms of ABA biosynthesis and signaling conservation were validated through functional genetic approaches. In barley, ABA content was positively correlated with *HvNCED2* expression during grain development, while a negative correlation was observed with *HvABA8′OH1* (Chono et al., 2006; Seiler et al., 2011). Moreover, increased *HvNCED1* expression and ABA content were observed under dormancy favoring environmental conditions, whereas non-dormant seeds show increased expression of ABA catabolism and GA biosynthesis genes (Gubler et al., 2008). In wheat, ectopic expressions of *TaNCED1*, *TaNCED2*, and *TaCYP707As* genes indicated their similar roles in regulating seed dormancy and germination (Son et al., 2016; Zhang et al., 2014). Expression levels of ABA biosynthesis and catabolism genes also complemented the germination differences between dormant and nondormant seeds in Brachypodium (Barrero et al., 2012). In addition to ABA metabolism genes, homologs of the ABA responsive gene *ABI3* are critical in regulating cereal grain dormancy. The rice *ABI3* homologue *VIVIPAROUS-1* (*OsVp1)* was reported to activate a major seed dormancy locus *SEED DORMANCY 4 (OsSdr4)* via the ABA signaling pathway (Chen et al., 2021; Sugimoto et al., 2010). Similarly, TaVp1 controls wheat grain dormancy and PHS resistance through interaction with ABI3-interacting protein (**Figure 3**) (Gao et al., 2014; Liu et al., 2024). Truncated transcripts of *OsVp1* and *TaVp1* have been associated with ABA sensitivities and PHS tolerances among various varieties and cultivars in rice and wheat, respectively (Fan et al., 2007; McKibbin et al., 2002; Utsugi et al., 2008).

Similar to ABA, sequence and functional conservation have also been reported for GA catabolism and biosynthesis genes. The rice Green Revolution gene *OsGA20ox2* was identified as the only candidate underlying the *Seed Dormancy1-2 (qSD1-2)* locus for endosperm-imposed dormancy and plant height (Ye et al., 2015). Similarly, the barley GA20-oxidase encoding gene has been identified as a candidate for a seed dormancy/pre-harvest sprouting locus by synteny analysis and genome wide association study (Li et al., 2004; Nagel et al., 2019). Enhanced seed dormancy was observed for mutants deficient in GA synthesis genes, including *OsGA20ox2*, *HvGA20ox2* and *HvGA3ox1* (Cheng et al., 2023; Xie et al., 2024; Ye et al., 2015), whereas advanced embryo germination and PHS was observed for a mutation in a rice GA catabolism gene *OsGA2ox9* (Xing et al., 2023). Like Arabidopsis, ABA/GA hormone balance also determines seed dormancy and germination in cereals (Tuan et al., 2018). A shift in ABA/GA hormone balance was reported to influence the induction and release of seed dormancy in barley and wheat (Gubler et al., 2008; Hoang et al., 2014). Furthermore, jasmonate and ethylene contribute to wheat seed dormancy release by modulating the ABA/GA balance (Cui et al., 2025; Nguyen et al., 2022; Sun et al., 2019). In rice, an AP2-type transcription factor OsAP2–39 directly controls the ABA synthesis gene *OsNCED1* and the GA deactivation gene *Elongation of Uppermost Internode (EUI)*, thus modulating the ABA/GA balance and related phenotypes including seed germination (Yaish et al., 2010). Several other genes, including *GRAIN NUMBER, PLANT HEIGHT AND HEADING DATE 7 (Ghd7), MOTHER OF FT AND TFL1 (MFT1) and MFT2, WEAK SEED DORMANCY 1 (WSD1)*, have been shown to regulate rice seed dormancy and germination through ABA/GA balance (Hu et al., 2021; Huang et al., 2023; Shen et al., 2024).

## 5 DOG1 is likely conserved but evidence is incomplete

*DELAY OF GERMINATION 1* (*DOG1*) was initially identified as a quantitative trait locus (QTL) for natural variation of dormancy in Arabidopsis (Bentsink et al., 2006). It encodes a protein that lacks domains with a known function. However, more recent studies revealed DOG1 is an α-helical protein that binds heme and interacts with ABA‐related PP2C phosphatases (Nishimura et al., 2018). In Arabidopsis, *AtDOG1* regulates dormancy induction, release, depth and dormancy cycling (Dekkers and Bentsink, 2015). It shows seed-specific expression and peaks at the late maturation stage (Bentsink et al., 2006). Inter-accession *AtDOG1* expression variation has been associated with seed maturation environment, while its transcript and protein levels positively correlate with seed dormancy levels in a temperature-dependent manner (Chiang et al., 2011; Nakabayashi et al., 2012). *AtDOG1* transcripts remain present in after-ripened seeds and disappear rapidly in both after-ripened and dormant seeds upon imbibition. Its protein localizes to the nucleus, and its abundance in freshly harvested seeds is highly correlated with the depth of dormancy. However, this correlation disappeared during after-ripening, which may be explained by possible protein modification, loss of self-binding or heme binding capacity (Nakabayashi et al., 2015, 2012; Nishimura et al., 2018).

As a central regulator of seed dormancy, ABA strongly influences the role of DOG1 in seed dormancy. Connections between AtDOG1 and ABA have been revealed at both the genetic and protein levels. The *dog1* loss-of-function mutant seeds show complete germination without after-ripening requirement and retain normal ABA sensitivity, while combination of *dog1* with GA biosynthesis mutant *ga1–3* or imbibition with GA biosynthesis inhibitor paclobutrazol inhibits germination (Bentsink et al., 2006; Nakabayashi et al., 2012). Both ABA and DOG1 are essential for the establishment of seed dormancy, as the absence of either one is sufficient to abolish dormancy. However, they seem to function in parallel or partially independent pathways, because increased levels of either one cannot compensate for the absence of the other, as evidenced by the *dog1 cyp707a2* mutant over-accumulating ABA and *aba1–1 DOG1-Cvi* combination harboring a strong *DOG1* allele (Bentsink et al., 2006; Nakabayashi et al., 2012). Double mutant analysis demonstrated that *dog1–1* enhanced the phenotype of the ABA insensitive mutant *abi3–1* during Arabidopsis seed development, implying genetic interaction between *AtDOG1* and *ABI3* (Dekkers et al., 2016). In line with this, AtDOG1 has also been shown to interact with ABA HYPERSENSITIVE GERMINATION1 (AtAHG1) and AtAHG3, core ABA signaling components at both protein and genetic levels during seed dormancy induction and after-ripening mediated dormancy release (Nee et al., 2017; Nishimura et al., 2018). While AtAHG3 was inhibited by PYR/PYL/RCAR receptors in the presence of ABA, AtAHG1 was resistant to such inhibition in the same experimental context (Antoni et al., 2012). This suggests the ability of AtAHG1 to regulate ABA signaling distinct from the canonical PYR/PYL/RCAR - ABA pathway, which explains the need of AtDOG1 to suppress PP2C activity completely, and hence corroborates the indispensable roles of both ABA and DOG1 for dormancy establishment (Nee et al., 2017; Nishimura et al., 2018). Recently, ABI5-binding proteins (AFPs) were identified as downstream targets of the DOG1-PP2Cs module, thus forming an AtDOG1-AtAHG1-AtAFPs route to regulate Arabidopsis seed dormancy (**Figure 3**) (Krüger et al., 2025; Nee et al., 2017; Nishimura et al., 2018), which orchestrates the fact that AtAFP2, one of the main DOG1-PP2Cs targets, has been implicated in breaking primary seed dormancy by progressively silencing *AtDOG1* (Deng et al., 2023). Although the seed dormancy phenotype of *abi5* mutants resembles that of the wild type (Finkelstein, 1994), ABI5 plays key roles in ABA-mediated inhibition of germination of non-dormant seeds and post-germination growth arrest (Lopez-Molina et al., 2001, 2002). Recently, it has been shown that ABI5 acts downstream of DOG1 in this specific process (Nishimura et al., 2025).

Through sequence similarity and functional genetic approaches, significant progress has been made in characterizing *DOG1 LIKE (DOGL)* genes in cereals. However, our understanding of their roles in seed dormancy is far from complete (**Table 2**). First of all, most reported functional analyses were conducted using ectopic expression of cereal *DOG1L*s in Arabidopsis or RNA interference mediated knockdown in cereals (Ashikawa et al., 2010, 2013; Ashikawa et al., 2014). It is anticipated that direct evidence would emerge from transgenic cereal lines or evolutionarily close model species such as Brachypodium (Scholthof et al., 2018). Moreover, *AtDOG1* encodes a protein with no similarity to known proteins. How DOG1 and its homologous protein function remain unknown, though different hypotheses have been proposed. Most notably, the properties of *AtDOG1* to undergo self-dimerization, bind heme, and interact with PP2Cs offer some clues to this puzzle, but more in-depth research would be required. Phylogenetic analyses indicate that *DOG1* family genes contain different clades or groups in both Arabidopsis and cereals, but only limited members have been characterized (Ashikawa et al., 2013; Nishiyama et al., 2021). The function and possible involvement of those untouched ones in dormancy remain unknown (**Figure 3**). Interestingly, recent evidence in wheat seems to support the conserved role of *DOG1Ls* in seed dormancy through the AHG-PP2C module (Yu et al., 2020; Zhang et al., 2025c, 2025). Research in Arabidopsis showed that this module is required at various stages, including seed dormancy induction and dormancy release by dry after-ripening, but information of this kind in cereals remains unexplored (Nee et al., 2017).

**Table 2:** Comparison of AtDOG1 and DOG1L genes in cereals.

Aspect	Arabidopsis	Temperate cereals	Rice
Gene identity	Five members, *DOG1* family genes *(DFGs)* (Nishiyama et al., 2021)	Four homologues	Three homologues
Genetic evidence	Loss-of-function mutants show non-dormant phenotype; ABA is indispensable for DOG1 function (Bentsink et al., 2006). DOG1 also regulates other seed developmental programs like storage accumulation and desiccation tolerance (Dekkers et al., 2016). The maternally imprinted gene *DOGL4* negatively affects seed dormancy and induces seed reserve accumulation (Sall et al., 2019; Zhu et al., 2018)	Ectopic expression of *TaDOG1Ls*, *HvDOG1Ls* in Arabidopsis enhanced seed dormancy, RNAi knockdown of *TaDOG1L4*, *HvDOG1L1* reduced seed dormancy (Ashikawa et al., 2013)	Gene swapping between different rice alleles (Wang et al., 2020b)
Allele variations	Natural variations observed, dormancy allele identified from a crossing between dormant and non-dormant accessions (Bentsink et al., 2010; Carlos Alonso-Blanco et al., 2003)	*HvDOG1L1* was found to play a minor role in a barley seed dormancy genome-wide association mapping (Nagel et al., 2019)	*OsDOG1L3* was proposed as a candidate gene for a rice seed dormancy QTL (Wang et al., 2020b)
Expression profile	Mainly expressed in the vascular tissue of the embryo, expression initiates after pollination until maturation (Bentsink et al., 2006; Nakabayashi et al., 2012)	Embryo specific expression observed in wheat dry seeds (Ashikawa et al., 2010)	Seed specific expression, expression initiates after pollination and peaked at 15th day post pollination (Wang et al., 2020b)
Hormonal regulation	Interaction with ABA signaling pathway through DOG1-AHG-AFP module (Nee et al., 2017; Nishimura et al., 2018); *dog1–1* mutant showed increased GA content when imbibed and decreased ABA content in dry seeds (Nakabayashi et al., 2012)	Interaction with ABA signaling pathway through TaDOG1L1/L4-TaPP2C-a6/a7/a10 module (Yu et al., 2020; Zhang et al., 2025c, 2025)	*OsDOG1L3* upregulated ABA-related gene expression and increased ABA content (Wang et al., 2020b)
Environmental regulation	Low temperature mediated dormancy induction, seed coat mediated dormancy release (Graeber et al., 2014; Kendall et al., 2011)	High temperature downregulated *TaDOG1* gene expression (Jiang et al., 2023)	/
Transcriptional regulation	Histone modification, alternative splicing, alternative polyadenylation, transcription elongation, and non-coding RNAs (Vollmeister et al., 2024)	/	/
Post-translational regulation	SUMOylation (Jing et al., 2024) and heme-binding (Nishimura et al., 2018)	/	/

## 6 The role of epigenetic mechanisms in seed dormancy

**Figure 4:**
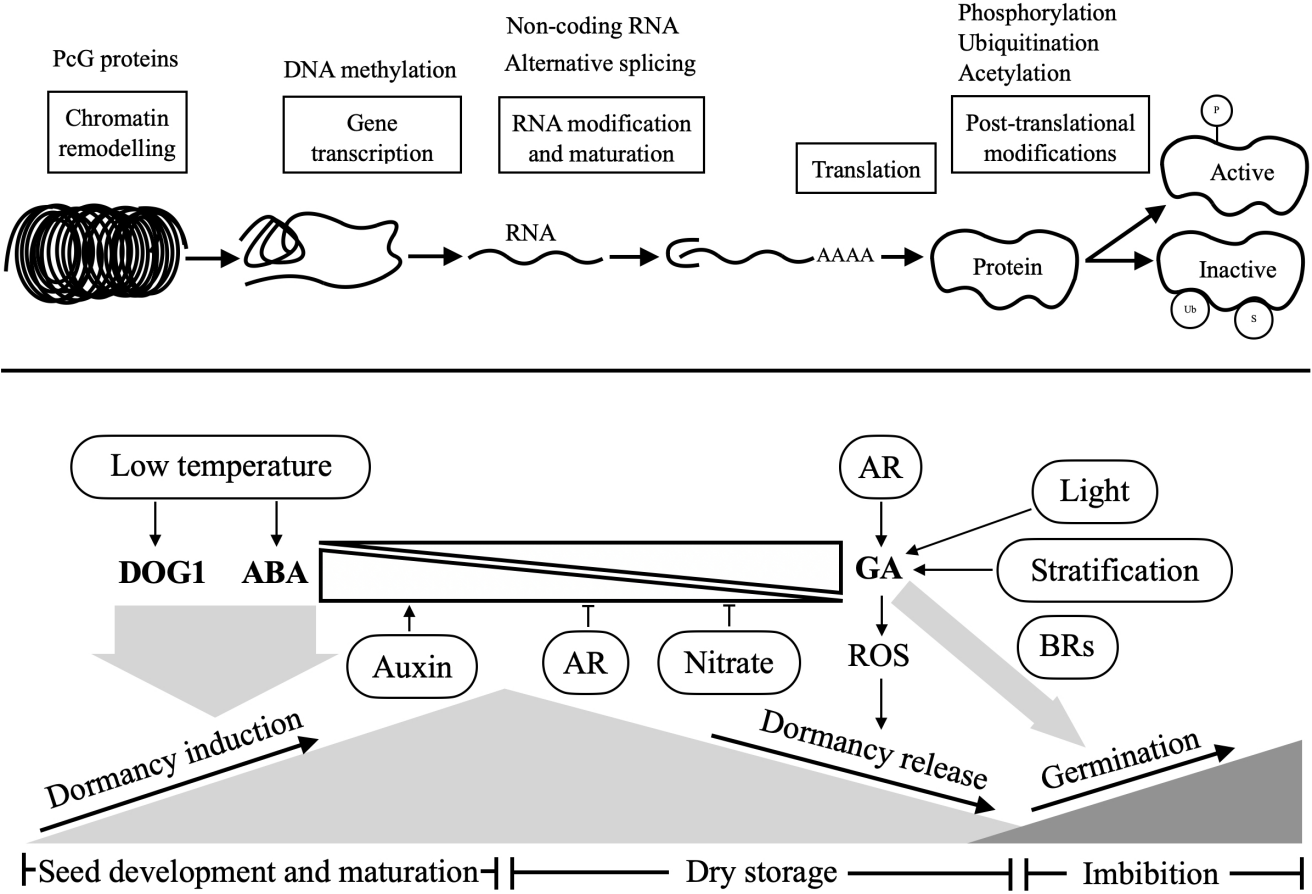
Environmental and hormonal regulation of seed dormancy and germination in Arabidopsis and associated genetic regulation processes. During seed development and maturation, low temperature could induce a strong seed dormancy level involving DOG1, while ABA is central to this induction. Dormancy can be released by either a period of dry storage (after-ripening) or cold stratification, which involves GA biosynthesis and accumulation. Upon imbibition, non-dormant seeds initiate germination stimulated by GA and promoted under light conditions. Besides environmental and hormonal regulations, seed dormancy and germination are tightly linked to genetic and epigenetic processes, including histone marks, gene transcription and translation, to protein post-translational modifications. The regulation processes were adapted from (Barrero et al., 2010). AR, after-ripening; BRs, brassiosteroids; ABA, abscisic acid; GA, gibberellic acid; DOG1, delay of germination 1; ROS, reactive oxygen species; PcG, polycomb group proteins.

Although seed dormancy has been extensively characterized at the genetic level, recent observations also reveal epigenetic regulation of this complex trait (Iwasaki et al., 2022; Tremblay and Qüesta, 2025). Through DNA methylation, chromatin remodeling and non-coding RNAs, epigenetic modifications regulate genomic imprinting, transcriptional gene silencing, developmental and environmental responses, thus contributing to coordinated seed development and fine-tuned seed dormancy (**Figure 4**).

### 6.1 DNA methylation

DNA methylation can contribute to silenced or activated gene expression depending on the sequence context, by adding a methyl group to the cytosine, especially at CAG, CHG and CHH, which modifies the chromatin structure and accessibility of transcription machinery. Typical enzymes involved can be DNA methylases and DNA demethylases. Key seed dormancy genes have been shown to be influenced by DNA methylation in Arabidopsis and cereals. For instance, a DNA demethylase REPRESSOR OF SILENCING1 (ROS1) plays a role in maternal imprinting of a germination-favoring gene *DOGL4* to negatively regulate seed dormancy in Arabidopsis (Zhu et al., 2018). Research on the rice *ROS1* gene seemed to support its conserved role in regulating genetic imprinting but gave no conclusion about its involvement in seed dormancy (Liu et al., 2018; Ono et al., 2012). DNA methylation of a nitrogen metabolism gene *ALLANTOINASE (ALN)* promoter can be stimulated by cold, leading to up-regulated seed dormancy. As loss-of-function of *ALN* could boost ABA production, it is thus not surprising that *ALN* repression promotes seed dormancy (Iwasaki et al., 2019; Piskurewicz et al., 2016; Watanabe et al., 2014). Methylome analysis of wheat germinating and dormant grains revealed increased differential methylation at *GA20ox*, *NCED*, *PP2C*, and *SnRK2*, major components of GA and ABA pathways (Zhang et al., 2021). Overall, current evidence supports the role of DNA methylation in regulating critical seed dormancy genes under diverse environments across developmental stages.

### 6.2 Chromatin remodeling

Chromatin organization in the nucleus influences DNA accessibility and gene transcription. Post-translational modifications (PTM) play important roles in modulating the DNA organization status through different groups of histone writers, readers and erasers. Among known histone PTMs, histone H3 methylation of lysine 27 (H3K27me) and H3K9me have been associated with transcriptional silencing. Histone mark writers for H3K27me and H3K9me include SUVH4/5/6 and Polycomb Repressive Complex 2 (PRC2), while erasers consist of Jumonji (JMJ)-domain containing proteins EARLY FLOWERING6 (ELF6) and RELATIVE OF EARLY FLOWERING 6 (REF6).

Initially, the PRC2 complex and its components were proven to be indispensable for coordinated seed development in both Arabidopsis and cereal crops through the manipulation of repressive histone marks. Mutants deficient in PRC2 members, including *FERTILIZATION INDEPENDENT ENDOSPERM (FIE)*, *EMBRYONIC FLOWER 1 (EMF1) and EMF2*, and *FERTILIZATION-INDEPENDENT SEED 2 (FIS2)*, show seed dormancy phenotypes (Bouyer et al., 2011; Luo et al., 2000, 1999). In the Arabidopsis *fie* mutants, the H3K27me3 deposition is abolished genome wide and seeds exhibit enhanced dormancy and germination defects, which was consistent with significant up-regulation of genes involved in seed maturation, including LEC2, ABI3, FUS3 and ABA signaling, most notably *ABI4* and *DOG1* (Bouyer et al., 2011). VERNALIZATION5/VIN3-LIKE 3 (VEL3), a PHD finger protein, and MULTICOPY SUPPRESSOR OF IRA1 (MSI1), a conserved subunit of PRC2, colocalize in the nucleolus and associate with a histone deacetylase complex to impose deacetylation and H3K27me3 mark in the central cell and retain them in mature seeds, thus regulating progeny seed dormancy. Interestingly, *vel3* mutant seeds set at 16 °C are non-dormant, suggesting that maternal maintenance of seed dormancy is under epigenetic influence (Chen et al., 2023a). Although the authors did not show the removal of these epigenetic marks when dormancy is broken, they proved the role of senescence associated gene *ORESERA1 (ORE1)* in seed dormancy. We can only speculate on the involvement of cellular localization under different environmental conditions. The flowering inhibitor VRN2 in association with PRC2 complex integrates environmental cues, including hypoxia, cold exposure and light into the epigenetic control of Arabidopsis development (Gibbs et al., 2018; Osborne et al., 2025). It would be tempting to explore the involvement of VRN2-PRC2 in the epigenetic regulation of seed dormancy as well.

In rice, chromatin remodeling has also been implicated in dormancy regulation. The *OsEMF2b* controls seed dormancy by regulating the expression of *OsVP1*. Downregulation of OsEMF2b expression was found to be in correlation with low expression of OsVP1 due to changes in both H3K27me3 and H3K4me3 enrichments (Chen et al., 2017). Another maternally expressed gene *OsFIE1*, regulates rice seed dormancy and aleurone development by depositing H3K27me3 marks on GA biosynthesis-related genes (Cheng et al., 2020). RNAi-mediated *OsFIE2* knockdown led to smaller seeds, partially filled seeds, and partial loss of seed dormancy, which is distinct from that of the Arabidopsis *fie* mutant (Nallamilli et al., 2013).

Apart from PRC2, Jumonji domain-containing proteins, which are capable of histone demethylation, control seed dormancy by influencing ABA and GA hormone balance in Arabidopsis. During germination, REF6 establishes an H3K27me3-depleted state, which facilitates the activation of hormone-related and expansin-coding genes. Chromatin occupancy of REF6 is gradually established during germination to antagonize increased PRC2 (Yan et al., 2018), thus contributing to robust seed germination and cell fate switches into vegetative development (Pan et al., 2023). In accordance with these observations, the *ref6* mutant seeds show enhanced dormancy due to increased endogenous ABA, which can be offset by overexpression of *CYP707A1* (Chen et al., 2020a). JMJ20 and JMJ22, two histone arginine demethylases, act redundantly to positively regulate seed germination through phytochrome B (PHYB). JMJ20/JMJ22 are repressed when PHYB is inactive, thus placing repressive histone methylations at GA synthesis genes *GA3ox1* and *GA3ox2*. Upon PHYB activation, de-repression of JMJ20/JMJ22 removes the repressive methylations, leading to increased GA levels and in turn promotes seed germination (Cho et al., 2012).

In cereals, JMJ proteins remove epigenetic marks and regulate germination through hormonal pathway components. In rice, OsJMJ718, which encodes a histone demethylase involved in H3K9 methylation, positively regulates rice seed germination. During seed imbibition, ABA content diminished while ethylene content was augmented, indicating that OsJMJ718 may influence seed germination through the ABA and ethylene signaling pathway (Jia et al., 2024). Another study in wheat showed that TaGATA1, a GATA transcription factor, recruits demethylase TaELF6-A1 to directly regulate *TaABI5* and enhances seed dormancy. TaGATA1 physically interacts with the putative demethylase TaELF6-A1, and TaELF6-A1 reduces methylation levels at the *TaABI5* promoter, which increases *TaABI5* expression and ABA signaling and enhances seed dormancy (Wei et al., 2023).

Besides the above-mentioned chromatin remodelers, a lot more components have already been reported, mostly in Arabidopsis, such as *HISTONE MONOUBIQUITINATION1 (HUB1)* underlying the *reduced dormancy 4* (*RDO4*) locus and histone deacetylases (HDAs) showing seed dormancy phenotypes. Several reviews can be referred to for further information (Ding et al., 2022; Nogueira do Amaral et al., 2024; Sato and Yamane, 2024; Tognacca and Botto, 2021).

### 6.3 Non-coding RNAs

Non-coding RNAs, including microRNA (miRNA) and long non-coding RNA (lncRNA), have been implicated in seed dormancy regulation in both Arabidopsis and cereals. miRNAs are produced from DNA and interact with the 3’ untranslated region of target mRNAs, leading to mRNA degradation and translational repression. During germination, Arabidopsis mutant seeds with a miR160-resistant form of AUXINRESPONSE FACTOR 10 exhibited hypersensitivity to ABA, whereas overexpression of miRNA160 resulted in reduced ABA sensitivity, indicating miR160 negatively regulates ABA sensitivity (Liu et al., 2007). Similarly, miRNA159-mediated cleavage of transcripts of two MYB transcription factors, *MYB33* and *MYB101* was found to mediate ABA hyposensitivity (Reyes and Chua, 2007). The major seed dormancy gene *DOG1* was shown to control seed dormancy and flowering time through the miRNA pathway in *Lactuca sativa* and Arabidopsis (Huo et al., 2016).

MicroRNAs have been indicated to regulate seed dormancy through ABA and GA pathways in cereal crops. In rice, mutations in one *MIR156* subfamily enhance seed dormancy and suppress PHS with minimal effects on important agronomic traits. Mechanistically, *mir156* mutations suppressed the GA synthesis pathway and increased rice seed dormancy (Miao et al., 2019). Another study of wheat-specific *miR9678* shows that this miRNA affects seed germination. Overexpression of miR9678 increased seed dormancy and PHS resistance associated with reduced bioactive GA levels, while silencing of *miR9678* leads to enhanced seed germination. *miR9678* targets a long noncoding RNA called WSGAR to induce the generation of phased small interfering RNAs, which interfere with seed germination. A positive correlation between *TaVP1* and *TaABI5* overexpression and increased endogenous *miR9678* levels was observed. Thus, *miR9678* influences seed germination via modulation of ABA/GA signaling (Guo et al., 2018).

Besides miRNA, several lncRNAs have been reported to regulate seed dormancy through DOG1 in Arabidopsis. Mechanistically, alternative splicing of *DOG1* produces five transcript variants: *DOG1α, β, γ, δ*, and *ϵ* (Nakabayashi et al., 2015; Nonogaki, 2017). Alternative splicing and alternative polyadenylation could on the one hand, contribute to the generation of the three major proteoforms DOG1α, DOG1β/γ/ϵ, and DOG1δ, and on the other hand give rise to long *DOG1 (lgDOG1)* harboring all three exons and short *DOG1 (shDOG1)* without the third exon (Cyrek et al., 2016). Later, a promoter for the transcription of an antisense transcript was identified in the 3’ region, including the third exon, which contributed to the 5’ capped and polyadenylated antisense transcript called *asDOG1.* As a negative regulator of *DOG1* expression, *asDOG1* inhibits seed dormancy and promotes germination. Disruption of *asDOG1* or removal of the *asDOG1* promoter led to significant upregulation of *DOG1* sense transcription (Fedak et al., 2016; Kowalczyk et al., 2017). Conservation of *asDOG1* across Brassicaceae species and the fact that it works in *cis* but not *trans* to regulate seed dormancy raises the question of whether this mechanism extends into other distantly related species, including temperate cereals. In addition to *asDOG1*, another lncRNA called *MUSHER* induces secondary dormancy via both DOG1 and ABA pathway. Through chromatin-localization, *MUSHER* promotes *DOG1* polyadenylation and expression of *PP2CA INTERACTING RING FINGER PROTEIN 1*, an E3 ligase which enhances ABA response, to adjust seed germination timing (Sacharowski et al., 2025).

In rice, a long non-coding RNA *VIVIpary* promotes seed dormancy release and pre-harvest sprouting by regulating ABA signaling. *VIVIpary* directly binds to the chromatin adaptor protein OsMSI1 and enhances its interaction with the histone deacetylase OsHDAC1, thus decreasing chromatin accessibility to adjust ABA signaling (Yang et al., 2025a). Transcriptome analysis comparing wheat germinating and dormant seeds highlighted the involvement of lncRNAs in transcriptional regulation of hormone biosynthesis and signaling pathways, including ABA, GA, ethylene and brassinosteroid (Zhang et al., 2021). These findings highlight the involvement of non-coding RNAs in modulating seed dormancy and germination, while also revealing potential opportunities for future efforts targeting PHS.

## 7 Environmental regulation of dormancy and germination

The switch to germinate or stay dormant is fundamentally determined by the ABA/GA ratio and the sensitivity to these hormones, which is under strong environmental influence. Environmental signals, including water, light, temperature, nutrients and oxygen, serve as major inputs for seed germination. These signals reflect seasonal variation, time of the year, depth in the soil, shade, time of the day, soil composition, allelopathic compounds and competition around. A great deal of knowledge is present for Arabidopsis, which shows ecological similarities to most temperate cereals. However, evaluating genetic conservation in temperate cereals and finding genetic switches to fine tune dormancy requires further investigation. The relevance of different environmental factors, their ecological significance, and genetics are discussed in this section.

### 7.1 The maternal environment as inducer of seed dormancy

The environment in which the mother plant grows can strongly affect seed dormancy at harvest (primary dormancy), and hence it is most relevant to pre-harvest sprouting resistance. In this section, different environmental factors, their impact on phytohormone regulation, their sensitivity and progeny seed dormancy are discussed.

#### 7.1.1 Temperature

As a major temporal signal, temperature is an important factor for setting dormancy levels. As annual species with a spring or winter habit, both Arabidopsis and temperate cereals experience similar seasonal temperature progression during their life cycles. Therefore, temperature similarly regulates their seed dormancy. Its depth at seed maturity is significantly influenced by the temperature experienced by the mother plant. Low temperatures during the pre-anthesis stage and grain development yield more dormant seeds, while high temperatures during seed development produce fewer dormant seeds in Arabidopsis and Brachypodium (Chen et al., 2014; Li et al., 2019). Arabidopsis and temperate cereal seeds usually mature during early summer, experiencing relatively cooler temperatures during seed development, hence producing dormant seeds. This dormancy helps seeds stay in the soil throughout the summer without the risk of untimely germination. Temperature affects seed dormancy through multi-layer signal integration and transduction, which involves seed coat modification (described in section 3), DOG1 and the central hormonal pathways. AtDOG1 protein levels were increased by low temperatures during seed maturation, and its levels in dry seeds determine dormancy depth. Inter-accession *DOG1* expression variation has been associated with seed maturation environment, most notably low temperature. Higher *DOG1* transcript and protein levels are detected in seeds matured at lower temperature (16/14 °C) compared to those matured at higher temperature (22/16 °C), corresponding to an increased seed dormancy level (Chiang et al., 2011; Nakabayashi et al., 2012). In Arabidopsis, *FLOWERING LOCUS C (FLC)* mediates low temperature induced seed dormancy through the ABA catabolism gene *CYP707A2* and GA synthesis gene *GA20ox1* (Chiang et al., 2009). Genetic interaction analysis between *FLOWERING LOCUS T (AtFT)*, *AtFLC*, and transcription factor *SHORT VEGETATIVE PHASE (SVP)* indicated that *AtSVP* functions upstream of *AtFT* and subsequently *AtFLC*, thus controlling Arabidopsis seed dormancy in a temperature-dependent manner (Chen and Penfield, 2018). Mutants with altered ABA or GA synthesis or signaling display reduced ability to enter low temperature induced deep dormancy (Kendall et al., 2011). In contrast, high temperature during seed maturation reduces seed dormancy in Arabidopsis, wheat, barley and rice, which involves different intermediates, including but not limited to *SPATULA (AtSPT)* and its homologue in rice and wheat called *SEED DORMANCY 6* (*OsSD6* and *TaSD6*), *PHYTOCHROME B (AtPHYB)*, *ABA-INDUCED WHEAT PLASMA MEMBRANE 19 (TaPM19)*, and *MOTHER OF FT AND TFL1 (AtMFT and TaMFT)* (Barrero et al., 2015; Donohue et al., 2007; Nakamura et al., 2011; Penfield et al., 2005; Wang et al., 2025; Xi et al., 2010; Xu et al., 2022). Altered ABA and GA hormonal balance and responsiveness were consistently associated with reduced seed dormancy in response to high temperature (Howard et al., 2012; Suriyasak et al., 2020; Tuan et al., 2021).

#### 7.1.2 Photoperiod

The environmental impact of maternal photoperiod on seed dormancy has been documented in *Arabidopsis thaliana* (Imaizumi et al., 2017; Munir et al., 2001; Penfield and Hall, 2009; Zha et al., 2020). Winter-annual Arabidopsis seeds matured under long days generally exhibit higher seed dormancy than those from short-day conditions. This is contradictory to temperature-dependent dormancy, as long days in nature are associated with warmer temperatures. This could be a preventive strategy for plants to avoid premature germination, as seeds that develop in the long day are more responsive to after-ripening for dormancy release (Imaizumi et al., 2017). Conversely, short maternal photoperiods enhance progeny responsiveness to stratification (quick switch for dormancy release), aiding the seeds developed in late summer to release dormancy quickly. By being more resistant to sudden cold patches, plants maintain seasonal resilience for germination. In contrast to Arabidopsis, the effect of photoperiod on bread wheat was not observed (Hickey et al., 2010, 2009). However, these results can be misleading as they use higher temperatures to grow plants, which may undermine dormancy induction. Although less is known about the underlying mechanisms involved in maternal photoperiod modulated dormancy in Arabidopsis or temperate cereals, one study suggests that impaired seed coat permeability caused by long maternal photoperiod in *Trigonella arabica* could be a possible mechanism (Gutterman, 1978).

#### 7.1.3 Other maternal factors

Progeny seed dormancy can also be influenced by the nutrient conditions the mother plants experienced. In different Arabidopsis ecotypes, plants grown under higher nitrogen produced less dormant seeds as compared to otherwise (He et al., 2014). Maternal environmental stresses could also affect progeny dormancy, as exemplified by parental drought stress, which produced more dormant seeds in wheat (Biddulph et al., 2005) and parental herbivory exposure leading to reduced Arabidopsis seed dormancy (Singh et al., 2017). As has been thoroughly reviewed, both water stress and biotic stress in the mother plant led to increased ABA production, which could be linked mechanistically to hormonal control of dormancy (Chen et al., 2020b).

Based on genotype and environmental interactions, the depth of dormancy is maintained at seed dispersal. Different genetic, physiological, environmental, spatial, and temporal factors contribute to the accumulation of ABA and other phytohormones to create a variable dormancy phenotype. At the time of seed dispersal, dormancy distribution is not always the same unless the plant is genetically non-dormant. The above-mentioned factors play an important role in plants to achieve this. This variation (bet-hedging) helps plants in the wild to get the most optimal moment to germinate, ensure the reach of the seed to longer distances, reduce offspring competition, and thus maximize the chance of survival for the next generation, and preserve the genetic information the seed carries (Gremer and Venable, 2014).

### 7.2 Environmental regulation of seed dormancy release

#### 7.2.1 Dormancy release by after-ripening

After dispersal in the soil, the seeds are subjected to after-ripening (AR), a long period of dry and warm storage that releases seed dormancy. In Arabidopsis, after-ripening can completely remove physiological dormancy from a few days to a few weeks, while in grasses and cereals, it can take up to a few months, maybe a year to completely remove dormancy, depending upon depth (**Table 1**). High temperature during AR reduces the time required to release dormancy and vice versa, which is highly relevant to PHS as revealed in wheat and barley (Gubler et al., 2005).

In Arabidopsis, an after-ripening treatment releases seed dormancy through modulating the hormone balance of ABA and GA. During after-ripening, ABA responsiveness, salicylic acid and ABA levels reduced, while GA and Jasmonic acid content increased (Ariizumi et al., 2013; Nelson et al., 2023; Yano et al., 2009). Changes of mRNA transcript and protein levels also occur during dry after-ripening, possibly through ROS, oxidation and irreversible carbonylation (El-Maarouf-Bouteau et al., 2013). It has been shown that targeted mRNA oxidation regulates sunflower seed dormancy alleviation during dry after-ripening (Bazin et al., 2011), in line with findings showing that after-ripening correlated with a progressive accumulation of ROS (Oracz et al., 2009). Besides oxidation, ROS plays its role in after-ripening through interaction with ABA signaling (Müller et al., 2009).

Dormancy release of cereal grains by after-ripening displays a consistent shift in hormone balance as observed in Arabidopsis, while also showing some differences, most notably in ABA signaling under imbibition conditions. Upon hydration of after-ripened barley grains, HvCYP707A1 and HvCYP707A2 initiated ABA catabolism (Chono et al., 2006; Gubler et al., 2008; Millar et al., 2006). This is accompanied by a decay of ABA signaling, performed by protein kinases such as SnRK2s (Ishikawa et al., 2019). Concomitantly, GA accumulation occurred, which corresponded to an increased expression of a GA biosynthesis gene, *HvGA3ox2*, and a catabolism gene, *HvGA2ox3*, creating a homeostasis (Gubler et al., 2008). During imbibition, ROS in barley embryo alleviated grain dormancy through activation of GA signaling and synthesis, which resulted from up-regulation of *HvGA20ox1* and a GA-induced gene *EXPANSIN* (*HvExpA11)* responsible for cell wall modification (Bahin et al., 2011). The role of ABA signaling in after-ripening is most pronounced in wheat. Transcripts related to ABA response were downregulated in after-ripened wheat grains under imbibition conditions, which involved oxidative modification of stored mRNAs and transcriptional response of *TaSnRK2s* and *TaABI5* (Gao et al., 2012, 2013; Liu et al., 2013a). Phytohormone interactions during after-ripening mediated wheat seed dormancy release revealed that auxin complements ABA to inhibit germination (Ramaih et al., 2003), while GA, jasmonate, brassinosteroid, ethylene, cytokinin and salicylic acid counteract ABA and promote germination (Chitnis et al., 2014; Jacobsen et al., 2013; Liu et al., 2013a; Nguyen et al., 2022).

Upstream mechanisms regulating AR remain largely unknown, although targeted oxidation of stored mRNA and protein, ROS mediated processes, and hormone changes may provide some hints for this enigma. It would be tempting to answer how seeds in a dry state sense and integrate environmental factors critical for the process. As the occurrence of PHS is tightly linked to a lack of dormancy around maturation, insights into this riddle could be highly informative and useful. Indeed, recent research began to reveal the role of a *MITOGEN-ACTIVATED PROTEIN KINASE KINASE 3* gene (*MKK3)* in after-ripening mediated grain dormancy release in wheat, barley and Arabidopsis (Otani et al., 2024). Association mapping in wheat revealed that *TaMKK3* confers PHS resistance by affecting the rate of dormancy loss during dry seed after-ripening (Shorinola et al., 2016; Torada et al., 2016). A similar observation was made for the wheat *MKK3* mutant *ENHANCE RESPONSE TO ABA8 (ERA8)*, showing enhanced grain dormancy and PHS resistance, which can be attributed to the altered GA and ABA sensitivity during after-ripening (Martinez et al., 2016). Moreover, one barley *MKK3* allele has been associated with a large loss of dormancy during grain after-ripening (Vetch et al., 2020). Of special interest, the biophysical boundaries confined by moisture content and storage temperature for dormancy release have been revealed in sunflower dry achenes, which provided long awaited experimental evidence about the modus operandi of this enigma (Arata et al., 2025). Future study would be anticipated to examine whether this is also the case in other seed plants, and how these biophysical conditions influence the after-ripening process mechanistically.

#### 7.2.2 Dormancy release by cold stratification

Cold stratification is the exposure of the imbibed seed to low temperature (2-5 °C), which serves as a signal for seeds to detect the chilling winter and prepare for germination afterwards. Ecologically, it might act as a fail-safe dormancy breaking mechanism for after-ripening, as the latter may not be fully adequate to break dormancy when the preceding summer did not saturate the after-ripening requirements. Cold stratification has been shown to increase germination in dormant seeds in several species, including Arabidopsis (Arc et al., 2012; Hauvermale et al., 2015). Simultaneously, cold stratification has also been reported to stimulate germination (Xu et al., 2016). An increase in GA content and sensitivity through the GA signaling gene *AtGID1* has been reported as a possible effect of stratification (Hauvermale et al., 2015). An increase in expression of the GA biosynthesis gene *AtGA3ox1* and reduced expression of the catabolism gene *AtGA2ox2* have been observed under cold imbibition. Moreover, the GA deficit mutant showed low sensitivity to cold stratification (Yamauchi et al., 2004). The upstream targets of *AtGA3ox1* under low temperatures are less known. However, association of *DOG1* negative regulation under low temperature can be a reason for upregulation of *AtGA3ox1* (Footitt et al., 2013). In addition, an increase in expression of the ABA catabolism gene *AtCYP707A1* downstream of C-REPEAT BINDING FACTORS in cold temperatures reduces ABA, while GA biosynthesis is enhanced. Cold imbibition also downregulates DELLA proteins, making seeds more sensitive to GA (Kendall et al., 2011). Moreover, the Arabidopsis brassinosteroid insensitive mutant *bri1–5* shows delayed germination and was insensitive to cold stratification, which can be reversed by inhibition of ABA biosynthesis. These findings imply that BR promotes cold-induced dormancy release through repressed ABA biosynthesis (Kim et al., 2019). The upstream roles of reported cold sensing genes like *FLC* and *FT* have not been reported to affect GA biosynthesis, but it is possible that they affect cold-induced dormancy breakage through ABA metabolism (Chen and Penfield, 2018).

In cereals, research has revealed the involvement of jasmonates in dormancy release. Xu, et al. showed an increase in jasmonates concentration and a decrease in ABA in response to cold in wheat (Xu et al., 2016). Blockage of jasmonate biosynthesis through acetylsalicylic acid makes seeds less sensitive to cold stratification and increases ABA concentration. They also show that germination in cold temperature was due to inhibition of ABA biosynthesis genes and increased expression of jasmonates biosynthesis genes. However, a recent study argued that jasmonates were more of an intermediate compound to maintain balance between ABA and GA and not directly involved in dormancy. Moreover, they reported that GA regulated dormancy release is independent of jasmonates (Nguyen et al., 2022).

#### 7.2.3 Nitric oxide pathways

Plants continuously seek favorable conditions for germination, and available nitrogen in the environment is a key component of such conditions, serving both as a macro-nutrient and a signal to break seed dormancy.

Exogenous applications of compounds such as sodium nitroprusside (SNP), potassium ferrocyanide, potassium ferricyanide, and potassium nitrate (KNO_3_) have been shown to alleviate Arabidopsis seed dormancy (Arc et al., 2013; Bethke et al., 2006; Matakiadis et al., 2009). Two independent studies demonstrated that this alleviation occurs through the ABA catabolism gene *AtCYP707A2*, because *cyp707a2* knockout seeds were insensitive to externally applied SNP and KNO_3_ (Liu et al., 2009; Matakiadis et al., 2009). NIN-like protein 8 (NLP8), a nitrogen modulated transcription factor, has been proposed as a master regulator of nitrate-promoted seed germination in Arabidopsis. On the one hand, NLP8 binds to the promoter region of *CYP707A2* to upregulate its expression under nitrate (Yan et al., 2016). On the other hand, NLP8 physically interacts with two critical downstream transcriptional regulators, ABI3 and ABI5, thus repressing ABA signaling during seed germination without affecting ABA content (Huang et al., 2025).

Additionally, post-translational modifications such as nitrosylation and tyrosine nitration facilitate seed dormancy release (Signorelli and Considine, 2018). Endogenous or exogenous NO inactivates the ABA receptor complex PYR/PYL/RCAR through tyrosine nitration, which subsequently activates PP2C (Castillo et al., 2015). The combined inactivation of SnRK2 kinases and nitrosylation of ABI5 results in its downregulation, lowering ABA sensitivity and increasing germination (Albertos et al., 2015). Another pathway by which NO effects ABI5 is through the degradation of a Group VII ethylene response transcription factor (ERF), a positive regulator of ABI5. In the presence of NO, ERF is degraded via the N-end rule pathway, ultimately leading to reduced ABI5 levels and decreased ABA content in Arabidopsis (Gibbs et al., 2014). The complete pathways of nitrogen regulated seed dormancy release are illustrated in **Figure 3**.

While our understanding of nitric oxide mediated seed dormancy release in temperate cereals is limited to the phenotypic level, the complex interplay of environmental and genetic factors suggests underlying mechanisms may be like those in Arabidopsis. Several studies have demonstrated the involvement of nitrates in breaking seed dormancy in various grasses. For example, research has shown that nitrates and NO enhance the germination of wheat, rice, and barley under different abiotic stresses (Duan et al., 2007; Parankusam et al., 2017; Shang et al., 2022; Zhang et al., 2023; Zheng et al., 2009). Additional studies have reported that nitrate and NO promote the release of dormancy in grasses such as wheat, barley, rice, wild oats, and some warm-season grasses (Bethke et al., 2004; Cohn et al., 1983; Hilton, 1985; Jacobsen et al., 2013; Ma et al., 2016; Matus-Cádiz and Hucl, 2003; Sarath et al., 2006; Zhang et al., 2005). In barley and wheat, research has more closely paralleled findings in Arabidopsis, with studies showing that exogenous NO application breaks dormancy; however, a mechanistic explanation has been confined to protein nitrosylation in response to NO application (Albertos et al., 2015; Ma et al., 2016; Sen, 2010).

#### 7.2.4 Nitrogen interplay with other factors

Nitrogen and nitric oxide have been known to interact with additional factors that affect seed dormancy, including ROS, light, and ethylene. It has been demonstrated that NO acts downstream of ROS to regulate ABA catabolism and GA biosynthesis (Liu et al., 2010). In Arabidopsis, exogenous application of nitrate donors reduced the dependence of seed germination on light via a cGMP (cyclic guanosine monophosphate)-mediated and phytochrome A-dependent mechanism (Batak et al., 2002; Teng et al., 2010). In addition to modulating ABA and GA pathways, NO also appears to influence the ethylene pathway, as evidenced by the positive correlation between NO levels and ethylene content in non-dormant seeds (Saini et al., 1985).

### 7.3 PHS resistance through delayed germination

Germination occurs under specific environmental conditions, and a lack of the optimum range can cause an inability to germinate without the presence of dormancy. Thus, delayed germination by narrowing the range of favorable environmental conditions offers an active switch to immediately germinate seeds without waiting for dormancy to be released, which could not only prevent PHS in the field but also give an industrially viable switch for rapid germination. For example, temperature-specific germination can be induced by tuning GA sensitivity, so that the temperature at harvest falls outside the conductive range of PHS, while a permissive range for germination can be applied when fast and uniform sprouting is desired. Another approach by engineering temperate cereal seeds to make them more sensitive to light could prevent sprouting on the spike, but allow immediate germination once buried in soil. Cumulatively, the seed remains fully viable but only germinates when a specific set of environmental cues overlaps, thereby reducing PHS risk without invoking dormancy. Different factors that affect germination are discussed in this section.

#### 7.3.1 Imbibition temperature

Beyond the regulation of seed dormancy during seed development, high temperature plays a crucial role in dormancy alleviation and germination after dispersal. When an imbibed seed is exposed to elevated temperatures, seed germination can be repressed, and this phenomenon is known as thermoinhibition. If high temperatures persist for a longer time, thermoinhibition can later convert into secondary dormancy mainly through *de novo* ABA synthesis and changes in ABA/GA sensitivity in the seed (Corbineau et al., 1988; Martel et al., 2018). Being winter annuals, both Arabidopsis and temperate cereals have highly conserved phenotypic responses to high imbibition temperature (Leymarie et al., 2009; Piskurewicz et al., 2023). High temperature incubation enhances ABA biosynthesis and GA catabolism gene expression in Arabidopsis wildtype seeds, but the same treatment promotes GA biosynthesis gene expression in ABA deficient mutant seeds. Conversely, in barley, ABA biosynthesis genes do not show any expression difference, but ABA catabolism gene expression levels reduce with high temperature incubation initially (Leymarie et al., 2009; Toh et al., 2008). Prolonged incubation at high temperatures ultimately results in *de novo* ABA synthesis in barley (Leymarie et al., 2008).

When Arabidopsis seeds are exposed to high temperatures, it activates various thermo-sensors (Casal et al., 2024; Yadav et al., 2025). These include heat shock proteins (HSPs), different phytochromes and cryptochromes, nitric oxide signaling proteins, and genes related to temperature regulated dormancy, such as *FLC, MFT*, and *DOG1* (Footitt et al., 2011, 2017; Piskurewicz et al., 2023). Among key regulators of secondary dormancy related to temperature, *DOG1* and *MFT* play essential roles. *DOG1* influences secondary dormancy by modulating ABA sensitivity rather than its content, indicating its role in ABA signaling rather than biosynthesis, and is consistent with the DOG1-PP2Cs interaction module (Footitt et al., 2015). This was further supported by the observation that one of the main targets of DOG1-PP2Cs, AFP2, has been shown to play a role in breaking secondary dormancy induced by high temperatures (Chang et al., 2018). *MFT* works alongside *DOG1* to maintain secondary dormancy in soil, with *MFT* being responsible for shallow response while *DOG1* regulates dormancy depth (Footitt et al., 2017). Furthermore, phytochromes provide additional flexibility in temperature-mediated dormancy. Phytochrome B downregulates the expression of ABA catabolism gene *CYP707A1* through PIFs (Piskurewicz et al., 2023), while phytochrome D is required to prevent secondary dormancy in response to high temperature by promoting GA accumulation through basic helix–loop–helix repressor *PIL5* (Martel et al., 2018). It is possible that phytochromes work antagonistically to create homeostasis, but there is no research supporting that argument. Similarly, the lack of reports in cereals likely suggests current limitations in available experimental data. However, genetic regulators influencing phytohormone sensitivity can be manipulated to modulate temperate cereals responses. These regulators, therefore, represent promising targets to fine-tune germination without necessarily enhancing primary dormancy.

#### 7.3.2 Light

For germination, light is typically required for Arabidopsis. Arabidopsis seeds respond strongly to red light promoting germination, while far-red reverses that effect, probably because small seeds use light as a cue for depth in the soil. By contrast, cereal grains often germinate in the dark (**Table 1**). Yet they also show better germination under red light, while blue and white light inhibit germination. Some people also believe that light to be a part of the normal physical environment necessary for germination and believe it has only a limited role in breaking seed dormancy itself (Barrero et al., 2012; Jacobsen et al., 2013).

Plant photoreceptors and their interacting proteins perceive and integrate light signals into the seed germination pathways. The Arabidopsis genome encodes five phytochromes (PhyA-E) to sense red/far-red light. Disruption of Arabidopsis phytochromes led to reduced germination, and *phyb* mutants exhibit the most pronounced phenotype (Arana et al., 2014; Dechaine et al., 2009; Donohue et al., 2008). PhyB acts upstream of two transcription factors, REVEILLE1 (RVE1) and RVE2, to repress red/far-red light reversible germination (Jiang et al., 2016). Besides light receptors, two PIFs have been shown to regulate germination in Arabidopsis. AtPIF6, which encodes a protein with a phytochrome binding domain, positively regulates germination (Penfield et al., 2010). In contrast, AtPIF4 interacts with AtABI4 to act as a transcriptional complex and promotes *NCED6* and *ABI4* expression, which leads to enhanced ABA biosynthesis and signaling (Luo et al., 2024). During Arabidopsis seed imbibition, AtPIF1 binds preferentially to the active forms of phytochromes and inhibits seed germination in darkness through modulating the GA and ABA hormone balance (Oh et al., 2004). Upon light exposure, active phytochromes induce AtPIF1 phosphorylation, which leads to its ubiquitination and subsequent degradation by the 26S proteasome (Shen et al., 2005; Zhu et al., 2015). Additionally, light sequesters AtPIF1 through interactions with AtHFR1, thus influencing genes associated with cell wall loosening, cell division, and hormonal pathways (Shi et al., 2013). Similar photo-reversible effect of red/far-red light on grain germination has also been revealed in Brachypodium, indicating the conserved role of phytochrome mediated seed germination in both plant groups (Barrero et al., 2012).

Blue light relieves seed dormancy and promotes germination in Arabidopsis (Stawska and Oracz, 2019), while it inhibits seed germination of dormant monocot grains, such as barley (Gubler et al., 2008; Hoang et al., 2014), wheat (Jacobsen et al., 2013) and Brachypodium (Barrero et al., 2012). A study of imbibed barley grains under blue light showed that this inhibition occurs through the ABA biosynthetic gene *HvNCED1* and *HvNCED2*, which led to increased ABA accumulation (Gubler et al., 2008; Hoang et al., 2014). The barley blue light receptor CRYPTOCHROME 1 (CRY1) was demonstrated to play a key role in perceiving and transducing blue light signals to regulate grain dormancy and germination (Barrero et al., 2014).

In summary, under a changing climate, a comprehensive understanding of environmental regulation of seed dormancy becomes very crucial (Duku et al., 2018; Marcinkowski and Piniewski, 2018). As prediction has shown that climate change may complicate the prospect of sprouting probability in dormancy-prone species (Shefferson et al., 2017), changing temperature and rainfall patterns during grain development and harvest may bring potential risks for PHS susceptible accessions. High temperatures during grain filling generally reduce the establishment of primary dormancy and may thus increase susceptibility to PHS. Moreover, an increase in rainfall and humidity close to harvest promotes premature imbibition of grains on the spike, thus raising the risk of PHS when dormancy levels are low. Eventually, historical dormancy thresholds for PHS resistance may become increasingly unreliable. Hence, in-depth information, particularly in temperate cereals, can help breeding programs to make informed decisions.

## 8 Current targets for genetic improvement of PHS resistance in cereal crops

Gene and pathway discovery has always been at the forefront for understanding any trait, let alone seed dormancy. Various forward and reverse genetic approaches have been employed to achieve this purpose. In the past, forward genetic approaches like quantitative trait loci (QTL) analysis, genome wide association studies (GWAS) and mutant screens coupled with next generation sequencing have been frequently applied. With the ease of genome editing techniques like CRISPR, validation of homologs from Arabidopsis in temperate cereals and genetic improvement of PHS resistance have been accelerated. Here are a few examples.

The conserved central mechanism of ABA and GA hormonal balance triggered active attempts to modulate grain dormancy through manipulation of endogenous hormone content or signaling in wheat, barley, sorghum and rice (Ban et al., 2022; Fu et al., 2022; Gubler et al., 2008; Rodríguez et al., 2025). As metabolism and signaling genes showed a strong impact on the phenotypes, including deficiency in plant growth, drought resistance and seed development, it will be difficult to use these genes as direct breeding targets for PHS resistance. Moreover, these phytohormones play huge roles in overall growth, development and life history of the plant, so modification in these genes just for PHS resistance can come with a cost. Although it is still very relevant to study these genes for identifying some ideal targets present in the pathways.

After unrevealing dormancy specific genetic pathways of these genes, and utilizing other approaches, we were able to identify certain targets specifically related to seed dormancy in temperate cereals (**Table 3**). Some of these genes can be used as a potential target for improved PHS resistance.

**Table 3:** Gene targets currently used to modulate seed dormancy in cereals.

Gene	Protein	Crop	Approach	Function	Background	Traits modulated	Other traits	Reference
*Tamyb10*	MYB domain protein 10	Wheat	CRISPR/Cas9	Flavonoid/phenylpropanoid metabolism	Fielder	Improved PHS tolerance	Convert white wheat into red	(Zhu et al., 2023)
*TaQsd1*	Alanine aminotransferase	Wheat	CRISPR/Cas9	Triple homozygous mutation delays grain germination	Fielder	Improved PHS tolerance	NA*	(Abe et al., 2019)
*TaSD6*	Seed Dormancy 6	Wheat	CRISPR/Cas9	Underlying natural variation of seed dormancy	Kenong199	Improved seed dormancy	Increased grain number per spike	(Xu et al., 2022)
*TaVP1*	Viviparous‐1	Wheat	CRISPR/Cas9	Homologous to Arabidopsis ABI3	Fielder	Decreased seed dormancy	NA	(Liu et al., 2024)
*TaSRO1*	Similar to RCD1	Wheat	CRISPR/Cas9	responsible for growth, development, and stress responses	Fielder	Enhanced seed dormancy	NA	(Liu et al., 2024)
*TaDOG1L4*	Delay of Germination 1	Wheat	RNA interference	Underlying natural variation in seed dormancy	Fielder	Decreased seed dormancy	NA	(Ashikawa et al., 2014)
*TaPHS1/TaMFT*	Phosphatidyl ethanolamine-binding protein	Wheat	RNA interference	Suppressor of grain germination	Bobwhite, Rio Blanco	Decreased seed dormancy	NA	(Liu et al., 2013b)
*HvGA20ox2*	Gibberellin 20-oxidase 2	Barley	CRISPR/Cas9	GA synthesis	Golden Promise	Improved seed dormancy	altered plant height	(Xie et al., 2024)
*HvGA3ox1*	Gibberellin 3-beta-dioxygenase 1	Barley	CRISPR/Cas9	GA biosynthesis	Vlamingh	Improved seed dormancy	optimized plant height and coleoptile length without adversely affecting other important agronomic traits	(Cheng et al., 2023)
*HvMPK6*	Mitogen-Activated Protein Kinase 6	Barley	CRISPR/Cas9	Functioning during embryo development and root development	Golden Promise	Reduced grain germination	NA	(Krenek et al., 2021)
*HvQsd1*	Alanine aminotransferase	Barley	CRISPR/Cas9	Regulates oxygen availability	Golden Promise	Improved seed dormancy	NA	(Hisano et al., 2022)
*HvQsd2*	Mitogen-Activated Protein Kinase Kinase 3	Barley	CRISPR/Cas9	Signal transduction cascade	Golden Promise	Improved seed dormancy	NA	(Hisano et al., 2022)
*HvABA8′OH1*	ABA 8’-hydroxylase 1	Barley	RNA interference	ABA catabolism	Golden Promise	Increased seed dormancy	NA	(Gubler et al., 2008)
*OsABA2*	Abscisic Acid2	Rice	CRISPR/Cas9	Activates the glucose signal, antagonizes the ethylene signal and promotes the synthesis of ABA	Yixiang1B	Reduced dormancy	NA	(Liao et al., 2018)
*OsABA8ox1*	Abscisic acid 8’-hydroxylase 1	Rice	CRISPR/Cas9	ABA catabolism	Ningjing6	strengthened seed dormancy	no effect on the yield	(Fu et al., 2022)
*OsABA8ox2*	Abscisic acid 8’-hydroxylase 2	Rice	CRISPR/Cas9	ABA catabolism	Ningjing6	strengthened seed dormancy	no effect on the yield	(Fu et al., 2022)
*OsABA8ox3*	Abscisic acid 8’-hydroxylase 3	Rice	CRISPR/Cas9	ABA catabolism	Ningjing6	strengthened seed dormancy	no effect on the yield	(Fu et al., 2022)
*OsGA2ox9*	Gibberellin 2-oxidase 9	Rice	CRISPR/Cas9	GA catabolism	Zhonghua 11	Decreased seed dormancy	less seed setting, longer panicles, and more branches of panicles	(Xing et al., 2023)
*OsGAP*	GTPase activating protein	Rice	CRISPR/Cas9	Increases ABA sensitivity in seed germination	Zhonghua 11	Enhanced seed dormancy	no difference in plant height, tiller number, grain shape and grain number with ZH11	(Xu et al., 2019)
*OsICE2*	Inducer of CBF Expression 2	Rice	CRISPR/Cas9	Regulate the ABA metabolism gene ABA8OX3 and NCED2	Zhonghua 11	Decreased seed dormancy	Reduced grain numbers per spike	(Xu et al., 2022)
*OsMAPK7*	Mitogen-activated protein kinase 7	Rice	CRISPR/Cas9	MKKK62-MKK3-MAPK7/MAPK14 module control seed dormancy by regulating the transcription of OsMFT	Zhonghua 11	Enhanced seed dormancy	NA	(Mao et al., 2019)
*OsMAPK14*	Mitogen-activated protein kinase 14	Rice	CRISPR/Cas9	MKKK62-MKK3-MAPK7/MAPK14 module control seed dormancy by regulating the transcription of OsMFT	Zhonghua 11	Enhanced seed dormancy	NA	(Mao et al., 2019)
*OsMKK3*	Mitogen-activated protein kinase kinase 3	Rice	CRISPR/Cas9	MKKK62-MKK3-MAPK7/MAPK14 module control seed dormancy by regulating the transcription of OsMFT	Zhonghua 11	Enhanced seed dormancy	NA	(Mao et al., 2024)
*OsMFT1*	Phosphatidyl ethanolamine-binding protein	Rice	CRISPR/Cas9	Regulate ABA and GA metabolism and their signaling pathways under salt stress	Zhonghua 11	Decreased seed dormancy	NA	(Lu et al., 2023)
*OsMFT2*	Phosphatidyl ethanolamine-binding protein	Rice	CRISPR/Cas9	Positively regulates ABA-responsive genes through interacting with OsbZIP23/66/72	Zhonghua 11	Decreased seed dormancy	NA	(Song et al., 2020)
*MIR156*	MIR156	Rice	CRISPR/Cas9	Suppress the GA pathway	Nipponbare, Xiuhua 134	Enhanced seed dormancy	negligible effects on shoot architecture and grain size	(Miao et al., 2019)
*OsNCED3*	Nine-cis-epoxycarotenoid dioxygenase 3	Rice	CRISPR/Cas9	Modulate ABA and GA levels in the embryo	Nipponbare	Decreased seed dormancy	decreased grain size and weight	(Chen et al., 2023b)
*OsSD6*	Basic-helix-loop-helix protein	Rice	CRISPR/Cas9	Influences key ABA synthesis and catabolism genes	Tianlong619, Wuyungeng27, Huaidao5	Enhanced seed dormancy	NA	(Xu et al., 2022)
*OsSdr4*	Seed dormancy 4	Rice	CRISPR/Cas9	A major quantitative trait locus for seed dormancy, encodes an unknown protein	Nipponbare	Decreased seed dormancy	No differences in grain length, grain width, grain thickness, 1000-grain weight, plant height, and tiller number	(Chen et al., 2021)
*OsVP1*	Viviparous-1	Rice	CRISPR/Cas9	Regulate key aspects of plant seed development and ABA signaling	Dongjin	Decreased seed dormancy	No significant differences in grain yield, straw weight, grain quality, and other main agronomic traits	(Jung et al., 2019)
*OsMODD*	Mediator of OsbZIP46 deactivation and degradation	Rice	CRISPR/Cas9	Inhibits the transcriptional activity of ABIs	Zhonghua 11	Increased seed dormancy	NA	(Guo et al., 2024)

*NA, not studied.

In barley, targeted mutagenesis in *Qsd1* (encodes an alanine aminotransferase, AlaAT) and *Qsd2 (*also called *MKK3)* revealed their essential roles in grain dormancy. In an Eastern Canadian barley biparental population LegCi, the non-dormant allele of *Qsd1* was associated with reduced hypoxia stress sensitivity, which promotes grain germination (Farquharson et al., 2025; Sato et al., 2016). As hypoxia has been known to increase barley embryo sensitivity to ABA and interfere with ABA metabolism (Benech-Arnold et al., 2006), it would be of practical importance to determine whether this mechanism extends into other barley accessions and temperate cereals. Both *qsd1*, *qsd2* single mutants and *qsd1/qsd2* double mutant showed delayed germination, and *qsd1* mutation partially suppressed the deep dormancy phenotype of *qsd2* mutants (Hisano et al., 2022). Similarly, CRISPR/Cas9-induced triple-recessive mutation in the wheat homologue of *Qsd1* resulted in a significantly deeper seed dormancy. In a field trial, the *TaQsd1* mutants showed variable seed dormancy phenotypes, depending on genetic background and environmental conditions. Mostly, with the high maternal temperature and PHS susceptible background, a moderate dormancy phenotype was observed. This makes *Qsd1* a controllable target for partial or complete loss of function to achieve an ideal PHS resistance phenotype.

Although no functional confirmation of *TaMKK3* has been reported in wheat, the association of PHS with natural or mutagen-induced alleles indicated its potential usefulness for PHS resistance (Jørgensen et al., 2025; Martinez et al., 2020; Nakamura, 2018; Zhang et al., 2025a). Research on rice and barley *MKK3* gene supports its conserved role as a negative dormancy regulator in cereals (Mao et al., 2019, 2024). As has been revealed to be involved in after-ripening, which is a controllable environmental switch (see section 7), the cereal *MKK3* genes could be one of the most interesting targets for conferring controllable PHS resistance. However, knowledge gaps exist concerning the exact mechanism and strong phenotype of the knockout mutants, limiting the usefulness of knockouts for direct application.

Apart from *MKK3*, *TaPHS1/TaMFT* has been revealed to be a critical positive regulator of wheat PHS resistance by independent studies (Liu et al., 2013b; Nakamura et al., 2011). While gene expression analysis linked this gene with low temperature induced grain dormancy, its RNAi-mediated knockdown mutant showed PHS phenotype (Liu et al., 2013b). The PHS resistant allele has been introduced into durum wheat and triticale for significantly suppressed grain germination (Kato et al., 2017; Moullet et al., 2022). Studies on the two rice *MFT* genes, *OsMFT1* and *OsMFT2*, reveal that the former promotes germination in the background of Nipponbare and Zhonghua 11, while the latter functions as a dormancy-promoter at least in the Zhonghua 11 cultivar, like *TaMFT* (Shen et al., 2024; Song et al., 2020; Zhang et al., 2025b). Intriguingly, another study in the Zhonghua 11 background found that *OsMFT1* promotes seed dormancy, but no effect of *OsMFT2* was observed (Lu et al., 2023). Thus, further investigation would be required to clarify the detailed role of *OsMFT* genes in regulating seed dormancy and germination in agronomically relevant genetic backgrounds under field conditions, which may pave the way for future application through overexpression or favorable allele stacking.

Another important target with potential for PHS resistance improvement could be *DOG1*. Its function and interactions with the environment and the hormonal pathway have been discussed in section 5. While potential interacting partners of *DOG1Ls* remain to be discovered in cereals, exploring other genes in this pathway could help to fine-tune seed dormancy levels. Moreover, despite the limited natural variation of *DOG1* reported in temperate cereals (Nagel et al., 2019), it would be tempting to screen for natural variation of *DOG1* using wheat landrace collection and identify functional alleles suitable for controlled dormancy (Pipatpongpinyo et al., 2020), which may serve as an interesting approach to address PHS under high seed development temperature. Another alternative could be DOG1 downstream factors, notably those involved in the DOG1-PP2Cs module. One compelling example could be the causal gene for a rice dormancy QTL *SDR3.1*, which encodes a mediator of OsbZIP46 deactivation and degradation (MODD) homologous to AtAFP acting downstream of the AtDOG1-AtAHG module (Guo et al., 2024).

Built on in-depth insights into the intricacy of seed dormancy and germination, pyramiding favorable alleles, genes, or QTLs would be feasible and worthwhile to achieve proper seed dormancy levels (Dong et al., 2025; Luo et al., 2021; Pipatpongpinyo et al., 2020; Wang et al., 2020a). At the same time, possible interactions between known dormancy regulators can be uncovered to find a more controllable dormancy switch. Additionally, the use of gene editing techniques to enhance or reduce the genetic expression of certain targets can be a helpful approach. As for pleiotropic trade-offs, targeting the most relevant domains and finding downstream targets in the context of PHS resistance is the way to go. Finally, to attain an ideal PHS phenotype, a model plant such as Brachypodium holds promise to fill the existing knowledge gaps.

## 9 Conclusion

Controlled seed dormancy in cultivated cereal varieties would be a sustainable solution to address pre-harvest sprouting. Conventional breeding and genetic modification could bring long-term solutions, especially in the context of a changing climate, which has been predicted to complicate the prospect of sprouting probability in dormancy-prone species.

Arabidopsis and temperate cereals contain several environmentally conserved mechanisms modulating seed dormancy, most notably the maternal effect, after-ripening and cold stratification, while only ABA/GA hormonal balance has been presented with consistent evidence supporting genetic conservation. However, obvious pleiotropic effects, namely its involvement in plant growth and development, make ABA/GA pathway a hard target to manipulate dormancy independently.

With respect to most of the environmental sensors and major dormancy regulators like *DOG1*, we still need to fill the remaining gaps concerning the genetic conservation between the two plant groups. In this context, Arabidopsis can serve as a template for initial hypothesis formation, but a robust and evolutionarily closer model system like Brachypodium could accelerate the efforts. Meanwhile, as a new frontier in crop breeding, we anticipate extensive investigation into the epigenetic regulation of seed dormancy and potential mitigation strategies for PHS. Another interesting avenue could be controllable resistance due to delayed germination rather than deep dormancy, which again warrants further in-depth study about the underlying intricacies of the environmental regulation aspects. We hope this manuscript can assist scientists in exploring untapped areas for effective PHS resistance.
